# TargetPrior: a miRNA-signature embedded evolutionary learning framework for prioritizing drug targets in acute myeloid leukemia

**DOI:** 10.1093/bioinformatics/btag635

**Published:** 2026-08-27

**Authors:** Ting-Yu Chen, Shinn-Ying Ho

**Affiliations:** Institute of Molecular Medicine and Bioengineering, National Yang Ming Chiao Tung University, Hsinchu, Taiwan; Institute of Molecular Medicine and Bioengineering, National Yang Ming Chiao Tung University, Hsinchu, Taiwan; Department of Biological Science and Technology, National Yang Ming Chiao Tung University, Hsinchu, Taiwan; Institute of Bioinformatics and Systems Biology, National Yang Ming Chiao Tung University, Hsinchu, Taiwan; Center for Intelligent Drug Systems and Smart Bio-devices (IDS^2^B), National Yang Ming Chiao Tung University, Hsinchu, Taiwan; College of Health Sciences, Kaohsiung Medical University, Kaohsiung, Taiwan

## Abstract

**Motivation:**

Prioritizing therapeutic targets from high-dimensional transcriptomic profiles is hindered by the underdetermined nature of the* p* ≫  *n* setting. While miRNA signatures can inform target prioritization, conventional accuracy-driven methods may yield unstable predictive signatures, reducing downstream network reliability and topology-guided candidate ranking.

**Results:**

We propose TargetPrior, a stability-aware evolutionary learning framework in which EL-CAML derives reproducible miRNA anchors from relapse-associated transcriptomic variation for candidate target prioritization. In childhood acute myeloid leukemia (CAML), EL-CAML identifies a parsimonious 18-miRNA continuous relapse-risk signature and 10 complementary stability-supported biomarkers, yielding 28 miRNAs for literature-curated miRNA–gene network construction. Repeated perturbation analysis supported the stability of high-frequency miRNAs, while analysis of the independent GSE196886 cell-sorted small RNA-seq dataset identified cell-population-specific expression differences. Benchmarking against an expanded set of clinically and biologically supported AML target references showed stronger early-rank retrieval than network-only and statistical approaches. TargetPrior is presented as a computational proof-of-concept for generating prioritized therapeutic hypotheses, rather than as a universal target-discovery solution.

**Availability:**

Code is available at: https://github.com/NYCU-ICLAB/TargetPrior and archived on Zenodo (DOI: 10.5281/zenodo.20394263).

## Graphical Abstract

**Graphical Abstract:**
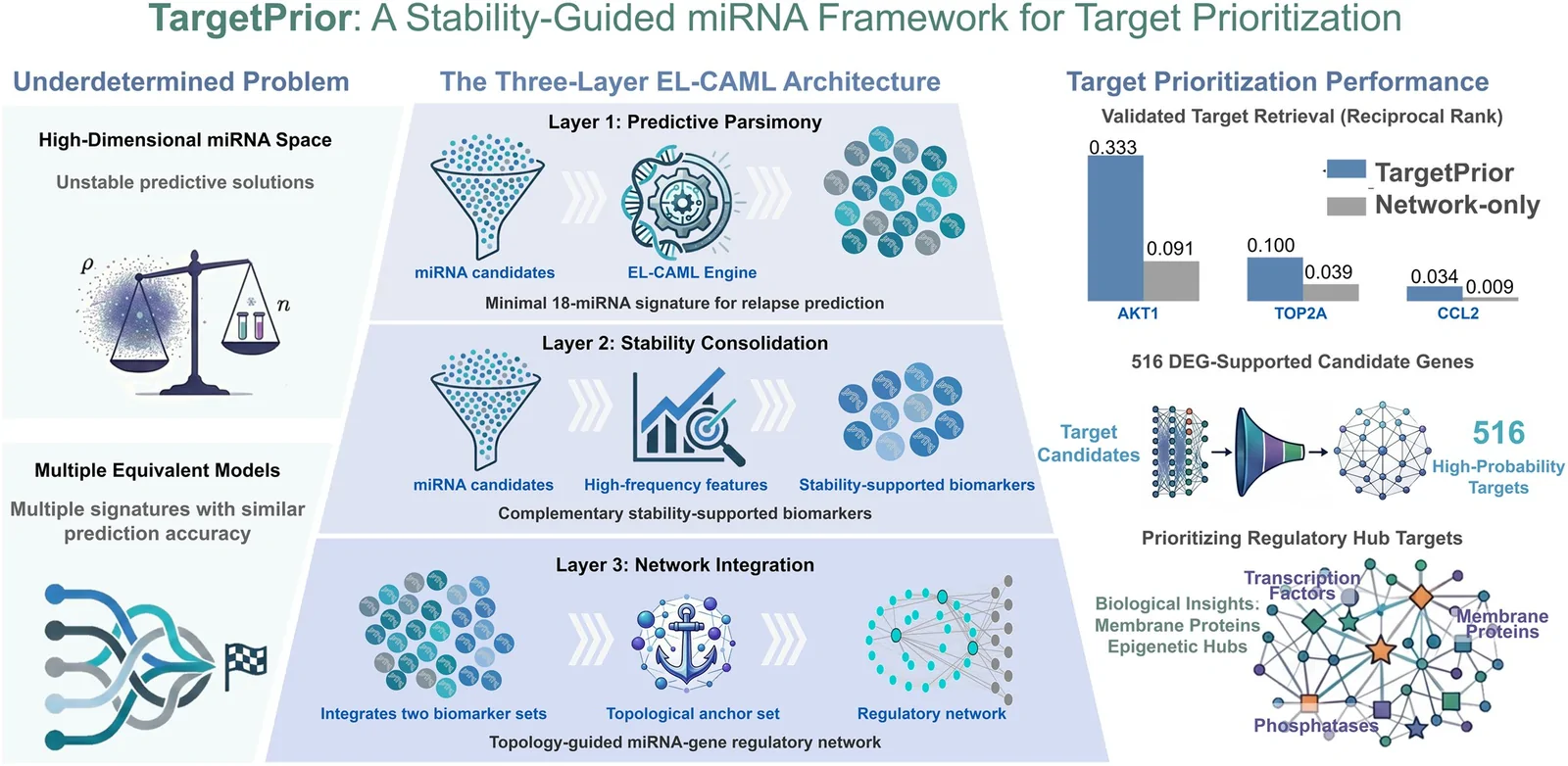
Graphical summary of the TargetPrior framework. High-dimensional miRNA data may produce multiple similarly predictive but unstable signatures. EL-CAML applies predictive parsimony, stability consolidation, and network integration to identify an 18-miRNA parsimony signature and 10 complementary stability-supported biomarkers, forming 28 topological anchors. These anchors constrain a literature-curated, DEG-supported miRNA–gene regulatory network containing 516 candidate genes. TargetPrior subsequently ranks network-central candidate genes and benchmarks the retrieval of AML-associated target references against a network-only baseline.

## 1 Introduction

Identifying therapeutically relevant targets remains a fundamental bottleneck in targeted cancer therapy (Mohs and Greig 2017, Tiwari *et al*. 2023). While high-dimensional transcriptomic profiles offer substantial opportunities for target discovery, conventional small-molecule pharmacotherapy is often constrained by the requirement for well-defined ligand-binding pockets (Gibbs 2000, Dang *et al*. 2017). Accordingly, next-generation modalities, including biologics and oligonucleotide therapeutics, have expanded the therapeutic focus toward traditionally difficult-to-drug regulatory proteins and network-central candidate nodes. These targets include transcription factors (TFs) and transcriptional cofactors (TCFs), phosphatases, protein–protein interaction interfaces, membrane proteins (MPs), transporters implicated in tumorigenesis, and immunomodulatory targets; many of these target classes lack the canonical binding pockets typically required for conventional small-molecule inhibition yet may participate in malignant progression (Blair 2021, Gulezian *et al*. 2021, Xie *et al*. 2023). As pleiotropic post-transcriptional regulators, microRNAs (miRNAs) can modulate multiple target transcripts (Crooke *et al*. 2018, Zhang *et al*. 2021) and participate in critical feedback loops involving tumor suppressors and oncogenes, such as TP53/miR-34 and MYC/miR-17–92 (Raver-Shapira *et al*. 2007, Jackstadt and Hermeking 2015). These regulatory properties provide a biological rationale for using miRNA–gene networks to prioritize candidate therapeutic targets, although computational prioritization requires systematic evaluation of feature-selection instability and literature-driven network bias.

However, the clinical therapeutic translation of transcriptomic target prioritization is hindered by the high-dimensional, small-sample-size (*p* ≫*n*) setting, particularly in molecularly heterogeneous diseases such as childhood acute myeloid leukemia (CAML) (Donoho 2006, Clarke *et al*. 2008). This ill-posed inverse problem creates the ‘Rashomon effect’, where many feature combinations yield near-equivalent predictive performance. Such model multiplicity may generate unstable signatures that capture sampling variation, batch effects, or platform-specific artifacts rather than reproducible disease-associated signals (Ioannidis 2005, Ein-Dor *et al*. 2006, Haibe-Kains *et al*. 2013). Conventional accuracy-driven strategies often select a single best-performing model from the solution space, overlooking statistically comparable alternatives and increasing the risk of limited cross-cohort generalizability (Breiman 2001). Therefore, feature stability should be interpreted as a computational reproducibility criterion, whereas biological causality and functional necessity require independent experimental validation.

Feature-selection instability also affects downstream systems biology analyses, particularly network-based target prioritization (Darrason 2018). Conventional network approaches typically rank differentially expressed genes (DEGs) directly using centrality indicators, such as closeness, betweenness, and maximum clique centrality (MCC) without explicitly modeling upstream feature-selection stability (Alam *et al*. 2022, Craig *et al*. 2024). However, DEG-only ranking can be sensitive to low-signal transcriptomic variation, while curated interaction databases may introduce literature-driven network bias because well-studied genes tend to accumulate more annotations and higher connectivity. Although standard feature selection methods such as LASSO and Boruta are widely used in high-dimensional biomarker discovery, single-model or wrapper-based selection procedures may return a single selected feature subset under a specified parameterization and may insufficiently explore alternative, statistically comparable solutions (Zou and Hastie 2005). Thus, establishing a stability-aware target prioritization framework requires moving beyond single-solution optimization to a strategy that leverages global combinatorial search to distill reproducible miRNA anchors from the underdetermined solution space.

To address this, we propose TargetPrior, a stability-aware framework that synergizes evolutionary learning (EL) with network biology for topology-guided candidate target prioritization. In this framework, relapse prediction is not treated as the final endpoint; instead, EL-CAML serves as an upstream computational engine that uses relapse-associated transcriptomic variation to derive stability-selected miRNA anchors. EL-CAML uses the inheritable bi-objective combinatorial genetic algorithm (IBCGA) (Ho *et al*. 2004), an optimization strategy previously applied in cancer biomarker discovery (Yerukala Sathipati *et al.* 2023a, 2023b, Yerukala Sathipati and Ho 2023, Sathipati *et al*. 2024). Instead of relying on a single-run heuristic, EL-CAML aggregates results from multiple independent runs to sample alternative solutions within the underdetermined solution space through a three-layer stability architecture based on repeated selection, consensus aggregation, and downstream network anchoring. Across independent evolutionary runs, EL-CAML identifies a parsimonious relapse-risk miRNA signature and complementary high-frequency biomarkers. These two feature sets are then combined into a unified topological anchor set for constructing a literature-curated, DEG-constrained miRNA–gene regulatory network. Thus, TargetPrior links continuous relapse-risk scoring to candidate target prioritization by using stability-selected miRNA features to guide downstream network construction and target ranking, not to serve as a standalone fixed-threshold classifier.

Despite advances in network medicine and machine learning (ML), computational target-prioritization studies often lack quantitative pharmacological benchmarking against curated therapeutic target reference sets (Morselli Gysi *et al*. 2021, You *et al*. 2022). We therefore applied TargetPrior to CAML as a computational proof-of-concept. TargetPrior prioritizes candidate genes that are connected to stability-selected, relapse-associated miRNA anchors within a literature-curated, DEG-constrained network. To quantitatively evaluate prioritization performance, we benchmarked the ranked candidates against an expanded AML target reference set compiled from clinically established and biologically supported sources and compared performance with network-only and statistical feature-selection baselines using early-rank retrieval metrics (Sherif *et al*. 2021, Zhang *et al*. 2021, 2022, Kuron *et al*. 2023, Ma *et al*. 2023, Xie *et al*. 2024, Röllig *et al.* 2025) (Supplementary Note 1, available as supplementary data at *Bioinformatics* online). In this design, EL-CAML generates continuous relapse-risk scores that preserve ordinal risk information for cross-cohort evaluation, while TargetPrior uses the associated miRNA anchors to support topology-guided therapeutic hypothesis generation. Accordingly, TargetPrior is presented as a computational proof-of-concept for prioritizing AML-relevant candidate target genes from transcriptomic datasets.

## 2 Methods

### 2.1 Data acquisition and preprocessing

**Figure 1:**
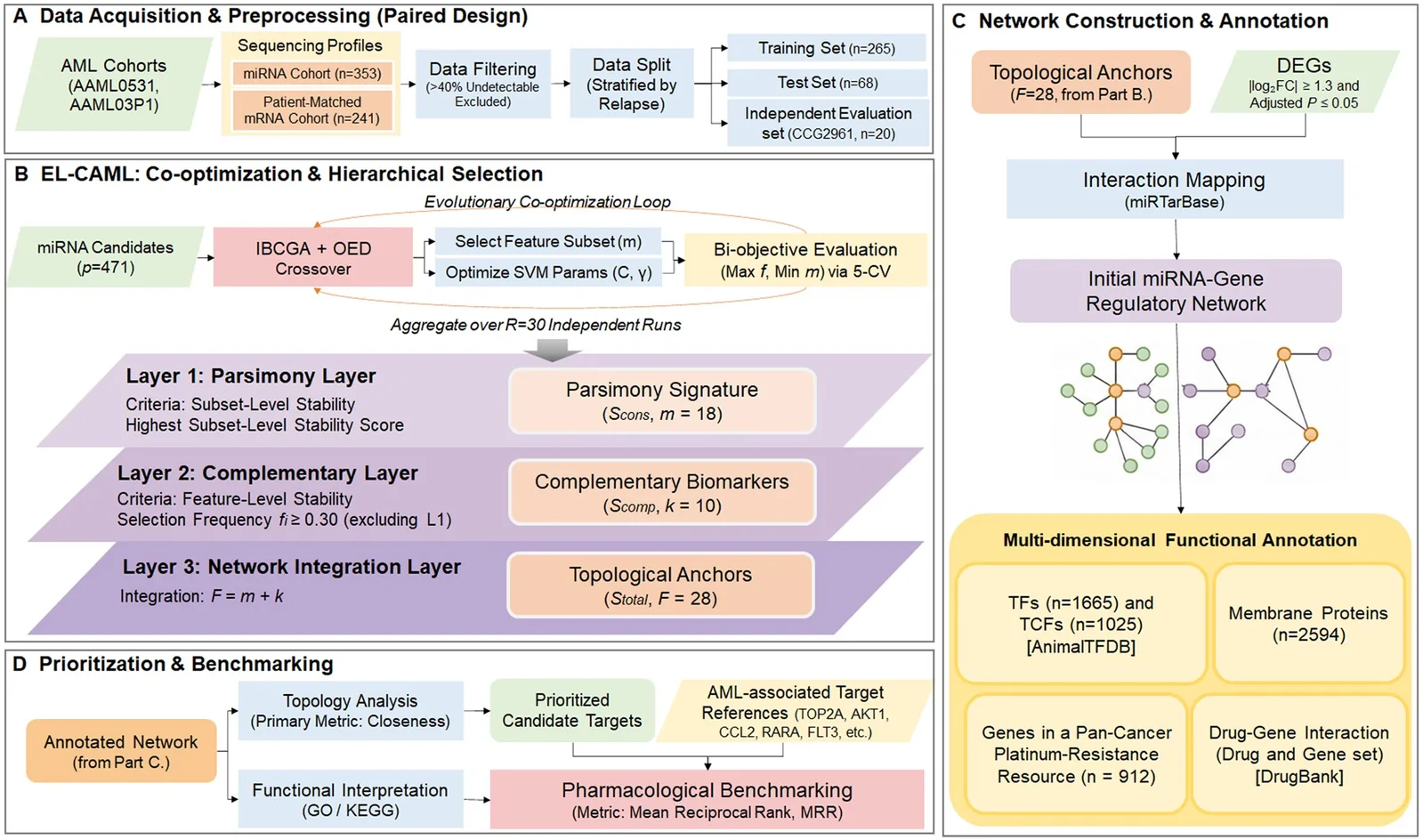
Overview of the TargetPrior framework. This evolutionary learning (EL)-embedded, miRNA-guided pipeline for drug target prioritization consists of four main modules: (A) data acquisition and transcriptomic preprocessing; (B) the EL-CAML module, performing feature-subset and SVM hyperparameter co-optimization and three-level selection to identify stability-selected miRNAs (establishing topological anchors); (C) miRNA-target regulatory network construction based on literature-curated interactions and multi-dimensional functional annotation; and (D) network-based target prioritization and pharmacological benchmarking, which utilizes network topology to rank therapeutic candidates and compares the ranked candidates against AML-associated target references. Abbreviations: TFs, Transcription factors; TCFs, Transcriptional cofactors.

miRNA expression profiles (e.g. transcripts per million) from diagnostic bone marrow samples were obtained from the TARGET-AML initiative (projects AAML0531 and AAML03P1) (Fig. 1A). To account for tissue-specific expression patterns and reduce the influence of low-abundance transcripts, features exhibiting undetectable expression across more than 40% of the cohort were systematically excluded. These filtered miRNA expression profiles were log_2_-transformed and partitioned into a training set (*n = *265) and an internal test set (*n = *68), stratified by relapse status. An independent cohort (CCG2961, *n = *20) was used as a proof-of-concept evaluation cohort. The combined CAML miRNA analysis set (*n = *353) comprised 157 relapsed and 196 non-relapsed cases (Table S1, available as supplementary data at *Bioinformatics* online). To prevent data leakage during predictive modeling, EL-CAML feature selection and SVM hyperparameter tuning were performed exclusively within the training set. For downstream DEG identification, patient identifiers in the miRNA analysis cohort were used to retrieve corresponding mRNA profiles. Only mRNA profiles from diagnostic bone marrow samples corresponding to patients in the miRNA cohort were retained. mRNA data were available for 241 of the 353 patients and were used for DEG identification and subsequent network construction. An independent cell-sorted pediatric AML small RNA-seq dataset (GSE196886, *n = *18) was used as an exploratory biological support dataset to evaluate compartment-specific expression profiles across leukemic and normal hematopoietic stem cell compartments (detailed in Supplementary Note 2, available as supplementary data at *Bioinformatics* online).

### 2.2 The TargetPrior framework and EL-CAML module

TargetPrior prioritizes candidate therapeutic targets by synergizing EL with network biology (Fig. 1B). Central to this framework is the EL-CAML module, which employs an IBCGA with orthogonal crossover (Ho *et al*. 2004) to solve the feature selection problem. The algorithm simultaneously optimizes two objectives: maximizing cross-validated predictive performance, defined as the Matthews correlation coefficient of a support vector machine (SVM) with five-fold cross-validation (5-CV), and minimizing the number of selected features *m*. The Matthews correlation coefficient is used as an internal optimization criterion for feature subset selection in the binary relapse-prediction task, whereas downstream prognostic interpretation is based on the continuous SVM-derived relapse-risk score and not on a universal fixed decision threshold. In addition, this EL process co-optimizes the selected feature subset and the SVM hyperparameters (the penalty parameter *C* and radial basis function kernel parameter γ). This mechanism allows each candidate feature subset to be evaluated under adaptively tuned hyperparameters, reducing dependence on suboptimal fixed hyperparameter configurations.

To improve reproducibility, the main IBCGA settings are summarized here and detailed fully in Supplementary Note 3, available as supplementary data at *Bioinformatics* online. In the current implementation, IBCGA uses a population size *N_pop_* = 50, *r_start_* = 30, *r_end_* = 10, crossover probability *P_c_* = 0.8, mutation probability *P_m_* = 0.05, maximum generations *G_max_* = 1000, and convergence patience *Convergence_max_* = 50. SVM hyperparameters *C* and *γ* are co-optimized within logarithmic search ranges. The search ranges are set from 10⁻¹ to 10³ for *C*, and from 10⁻⁴ to 10¹ for γ. Because evolutionary optimization may yield run-to-run variability even under fixed implementation settings, feature selection is repeated across *R *= 30 independent evolutionary runs. The final miRNA signature is then defined by consensus aggregation, retaining features that are repeatedly selected across independent runs as stability-selected candidate miRNA anchors for downstream network construction.

### 2.3 Stability-aware miRNA anchor definition

EL-CAML implements a stability-driven global search strategy based on *R* independent runs. For each miRNA feature *i*, the feature-level selection frequency *f_i_* estimates the empirical selection probability across the independent runs:


$$f_{i}=\frac{1}{R}\sum_{r=1}^{R}I_{r,\;i}$$

where $I_{r,\;i}\in \{0,1\}$ indicates whether feature *i* is selected in the optimal model from run *r*. EL-CAML then employs a three-layer stability architecture. Layer 1, the parsimony layer, identifies the consensus parsimony signature (denoted as *S_cons_*). For a subset *S* with *m = |S|*, the subset-level stability score *SSS(S)* is computed as the mean selection frequency of its constituent features:


$$\mathrm{SSS}(S)=\frac{1}{m}\sum_{i\in S}f_{i}$$

The final parsimony signature is selected as $S_{\mathrm{cons}}=\arg\underset{S}{\max}\mathrm{SSS}(S)$, representing the subset with the highest subset-level selection reproducibility among candidate solutions. Layer 2, the complementary layer, retrieves the ‘complementary biomarkers’ (*S_comp_*) from high-frequency features not already included in *S_cons_*. Features satisfying *f_i_* ≥ *θ* are retained, whereas features with *f_i_* < *θ* are excluded, ensuring a mutually exclusive extraction (*S_cons_* ∩  *S_comp_* = ∅). This stability criterion is used as a computational reproducibility filter and does not independently establish biological relevance or causal involvement. Layer 3, the network integration layer, integrates these two sets into a unified topological anchor set (*S_total_* = *S_cons_* ∪  *S_comp_*), with a size of *F *=* m *+* k* (where *k = |S_comp_|*). In the current CAML case study, *R *= 30, *m *= 18, *θ*  =  0.30, *k *= 10, and *F *= 28. Thus, EL-CAML links binary relapse prediction to candidate target prioritization by using stability-selected miRNA features as topology-guiding anchors, not as a standalone prediction endpoint.

### 2.4 Continuous relapse-risk scoring and predictive evaluation

Following model training, SVM decision values are transformed into uncalibrated continuous relapse-risk scores bounded within [0, 1] using a monotonic piecewise linear scaling function: a decision value of 0 is mapped to 0.5, while negative and positive values are linearly scaled to [0, 0.5] and [0.5, 1], respectively (as detailed in Supplementary Note 4, available as supplementary data at *Bioinformatics* online). The value 0.5 therefore represents the rescaled SVM decision boundary and should not be interpreted as a universal clinical threshold. Instead, the transformed score is used to preserve the ordinal ranking induced by the SVM decision function across patients. The association between score-defined risk strata and overall survival was assessed using Kaplan–Meier (KM) survival analysis and the log-rank test.

To assess whether the EL-derived anchor set captured network-level information beyond feature-set size or marginal statistical ranking, we compared *S_total_* with two baselines: a size-matched random miRNA selection control and a Wilcoxon-ranked statistical filtering benchmark. For the random control, 30 independent sets of *F* miRNAs were sampled without replacement from the *P *= 471 candidate miRNAs. Each random set was processed using the same miRTarBase interaction mapping and DEG-filtering procedure as *S_total_*, and the number of unique downstream DEG targets was recorded at each |log_2_FC| threshold. The statistical benchmark comprised the top *F* miRNAs ranked by Wilcoxon *P*-values (Supplementary Note 5, available as supplementary data at *Bioinformatics* online). These comparisons evaluated whether the EL-derived anchors were associated with stronger downstream network connectivity and DEG enrichment than baseline feature-selection strategies.

To benchmark the predictive performance of the ‘parsimony signature’ (denoted as *S_cons_* with size *m*), EL-CAML was compared with a suite of binary ML prediction models and feature-selection strategies under the same 5-CV framework. Threshold-dependent metrics were reported for comparability with conventional binary relapse prediction, whereas AUC was used to evaluate threshold-independent discrimination based on model-derived continuous scores. Comparative models included multilayer perceptron (MLP), random forest (RF), eXtreme Gradient Boosting (XGBoost), gradient boosting (GB), and AdaBoost. Size-matched feature-selection baselines included Wilcoxon statistical filtering, cross-validated LASSO, and Boruta. Detailed hyperparameter settings and implementation frameworks are provided in Supplementary Note 6, available as supplementary data at *Bioinformatics* online. In the current CAML case study, the baseline simulation was repeated *N_rep_* = 30 times and the size-matched baselines were constrained to *m *= 18 features.

### 2.6 Network construction and drug target annotation

To systematically map regulatory links from the topological anchors (*S_total_*) to potential therapeutic targets, we constructed a miRNA–gene regulatory network (Fig. 1C). DEGs between relapsed and non-relapsed CAML cases were identified using the limma R package in the patient-matched mRNA cohort (*n = *241). Filtering thresholds were defined as |log_2_ fold change (FC)| ≥ *τ_fc_* and adjusted *P *≤ 0.05. Sensitivity analysis supported the use of *τfc *= 1.3 as a threshold (Fig. S1, available as supplementary data at *Bioinformatics* online) that reduces network density by filtering out marginal low-effect expression changes while retaining higher-confidence dysregulated genes (Menche *et al*. 2015). The regulatory network was assembled by retaining miRTarBase-curated miRNA-target interactions in which target genes were also present in the identified DEG pool. Thus, network edges were literature-curated and constrained by cohort-level differential expression evidence.

Network nodes were functionally annotated using multiple resources. TFs and TCFs were annotated via AnimalTFDB 3.0 (Hu *et al*. 2019); MPs were identified using a curated actionable surfaceome dataset (Lin *et al*. 2019); and network genes were additionally annotated using a literature-curated pan-cancer platinum resistance (PR) resource to examine resistance-associated functional processes (Huang *et al*. 2021). This annotation was not used for candidate ranking. Drug–gene interaction data from DrugBank 5.0 were integrated for drug-target annotation (Wishart *et al*. 2018). Kyoto Encyclopedia of Genes and Genomes (KEGG) pathway and Gene Ontology (GO) enrichment analyses were performed for functional interpretation of prioritized candidate genes using adjusted *P *≤ 0.05.

### 2.7 Network-based prioritization and benchmarking

To quantitatively evaluate the target prioritization capability, we performed pharmacological benchmarking against a curated AML target reference set (Fig. 1D; Supplementary Note 1, available as supplementary data at *Bioinformatics* online). This reference set comprised nine AML-associated target references spanning clinically established, pharmacologically investigated, and biologically supported evidence tiers: *AKT1*, *TOP2A*, *CCL2*, *RARA*, *FLT3*, *BCL2L2*, *MEN1*, *MCL1*, and *MDM2*. Multiple centrality metrics, including MCC, betweenness, degree, and closeness, were computed to characterize network topology. For benchmarking, candidate genes were ranked using closeness centrality as the primary ranking metric because it quantifies how proximally a candidate node is positioned relative to other nodes within the constrained regulatory network, thereby reflecting potential network-level reachability of perturbation. To reduce dependence on a single topological metric, betweenness, degree, and MCC were analyzed as sensitivity metrics, and their results are reported in Table S2, available as supplementary data at *Bioinformatics* online. Target-ranking performance was quantified using early-rank retrieval metrics and enrichment-based statistical tests. Complete results for Precision@K, Recall@K, mean reciprocal rank (MRR), the hypergeometric test, the Mann–Whitney *U* test, and 1000-run degree-quantile-matched permutation analyses are provided in Supplementary Note 7, available as supplementary data at *Bioinformatics* online.

To isolate the incremental contribution of the EL-derived anchors, TargetPrior was evaluated against four alternative prioritization strategies: (1) a network-only baseline that ranked candidate genes directly from the DEG pool without miRNA-guided constraints; (2) a Wilcoxon-ranked univariate statistical strategy that used the top *F* miRNAs ranked by raw *P*-values as alternative anchors; (3) a LASSO-regularized strategy that used a size-matched feature subset of *F* miRNAs selected by cross-validated L1-regularized logistic regression as alternative anchors; and (4) a broad screening strategy using a broader statistically significant feature set (denoted as *F_broad_*). This comparative design evaluated whether stability-aware evolutionary feature refinement improved early retrieval of AML-associated target references within the constructed regulatory network. In the current study, the specific parameters were *F *= 28 and *F_broad_* = 179.

## 3 Results

### 3.1 Identification of a parsimony signature for continuous relapse-risk prediction

The EL-CAML engine, functioning as the upstream feature-selection module of TargetPrior, addressed the curse of dimensionality inherent in high-throughput transcriptomic profiles by coupling global combinatorial search with stability-aware selection. This process effectively balanced the stability–performance trade-off through a multi-layer stability architecture. Within the parsimony layer, EL-CAML identified an 18-miRNA parsimony signature (*S_cons_*) associated with relapse-risk stratification (Table 1). Saturation analysis (Supplementary Figs. S2 and S3, available as supplementary data at *Bioinformatics* online) indicated that this subset corresponds to a stability–performance inflection point, supporting its use as a compact continuous risk-scoring signature.

**Table 1:** Summary of the 18-miRNA parsimony signature (*S_cons_*).

Rank	miRNA	MED (ΔAccuracy%)	Diagnostic prediction (AUC)	Relapse prediction (AUC)	TargetPrior Role	Pan-cancer role	AML-related role	Drug resistance	Wilcoxon *P*-value	Log-rank *P*-value
1	miR-27a	4.351	0.76 ± 0.04	0.55 ± 0.02	tsmiR	oncomiR	tsmiR	Yes	<0.001	0.1874
2	miR-1271	2.849	0.72 ± 0.05	0.56 ± 0.02	tsmiR	tsmiR	tsmiR	–	0.12	0.0095
3	miR-106b	2.628	0.84 ± 0.03	0.60 ± 0.03	oncomiR	oncomiR	oncomiR	Yes	<0.05	<0.001
4	miR-3607	2.186	0.79 ± 0.05	0.57 ± 0.03	tsmiR	tsmiR	–	–	<0.05	<0.001
5	miR-6874	1.966	0.71 ± 0.05	0.55 ± 0.02	tsmiR	novel	novel	novel	<0.05	<0.001
6	miR-3605	1.877	0.73 ± 0.04	0.55 ± 0.03	tsmiR	tsmiRs	not clear	–	0.197	<0.001
7	miR-99b	1.656	0.79 ± 0.04	0.57 ± 0.02	oncomiR	oncomiR/tsmiR	–	Yes	0.063	0.114
8	let-7g	1.524	0.79 ± 0.04	0.57 ± 0.03	tsmiR	tsmiR	tsmiR	Yes	<0.01	<0.05
9	miR-335	1.391	0.83 ± 0.04	0.57 ± 0.02	oncomiR	oncomiR/tsmiR	oncomiR/tsmiR	Yes	<0.005	<0.05
10	miR-133a-1	1.391	0.71 ± 0.04	0.56 ± 0.03	tsmiR	tsmiR	tsmiR	Yes	0.101	0.081
11	miR-186	1.259	0.79 ± 0.04	0.59 ± 0.02	tsmiR	tsmiR	tsmiR	Yes	0.058	0.087
12	miR-1343	1.259	0.76 ± 0.04	0.54 ± 0.03	tsmiR	oncomiR/tsmiR	–	–	<0.05	<0.001
13	miR-589	1.038	0.77 ± 0.04	0.55 ± 0.02	tsmiR	oncomiR	oncomiR	Yes	0.066	0.163
14	miR-937	0.95	0.81 ± 0.04	0.57 ± 0.03	oncomiR	oncomiR/tsmiR	–	–	0.183	<0.001
15	miR-6842	0.817	0.76 ± 0.04	0.58 ± 0.03	oncomiR	oncomiR	–	–	<0.05	0.248
16	miR-1538	0.685	0.74 ± 0.05	0.56 ± 0.02	tsmiR	oncomiR/tsmiR	–	–	0.154	<0.001
17	miR-100	0.331	0.70 ± 0.05	0.57 ± 0.02	oncomiR	oncomiR/tsmiR	oncomiR in adult	Yes	0.079	<0.001
18	miR-1307	0.066	0.72 ± 0.04	0.57 ± 0.02	tsmiR	oncomiR/tsmiR	–	Yes	0.134	<0.05

The miRNAs are ranked according to their main effect difference (MED) scores (expressed as ΔAccuracy%), which quantify each feature’s individual contribution to binary relapse-prediction performance during model optimization. Relapse-risk discrimination was evaluated using AUC, which reflects threshold-independent separation of relapsed versus non-relapsed cases based on continuous model scores. Statistical significance of expression differences between relapse and non-relapse groups was assessed using the Wilcoxon rank-sum test. Survival associations were evaluated utilizing the log-rank test in Kaplan–Meier (KM) survival analysis. Abbreviations: AUC, area under the receiver operating characteristic curve; oncomiR, oncogenic miRNA; tsmiR, tumor-suppressor miRNA.

Feature importance analysis using main effect difference (MED) scores (Yerukala Sathipati and Ho 2017) identified miR-27a (MED = 4.351) as the highest-ranked contributor, followed by miR-1271 and miR-106b. These miRNAs were interpreted as high-priority predictive features and not as individually validated causal regulators. Given the limited size of the independent proof-of-concept evaluation cohort, we quantified performance uncertainty using nonparametric bootstrap resampling with 1000 iterations. The EL-CAML model achieved AUC values of 0.93 (training), 0.88 (5-CV), 0.76 (internal test), and 0.80 ± 0.018 (independent cohort evaluation), indicating retained threshold-independent discrimination across evaluation settings. For conventional binary-classification reporting, the corresponding threshold-dependent MCC values were 0.76, 0.75, 0.28, and 0.34 ± 0.038, respectively. As a sensitivity analysis of fixed-threshold performance, post hoc threshold optimization and probability recalibration were evaluated (Supplementary Note 4, available as supplementary data at *Bioinformatics* online). These analyses showed that fixed-threshold classification was sensitive to inter-cohort distribution shifts and was not fully corrected by global recalibration methods, including Platt scaling and isotonic regression. In contrast, the continuous EL-CAML risk score preserved ordinal risk information, as supported by threshold-independent metrics (Fig. S4, available as supplementary data at *Bioinformatics* online). Accordingly, EL-CAML is best interpreted as a continuous relapse-risk ranking model, not as a fixed-threshold classifier.

**Figure 2:**
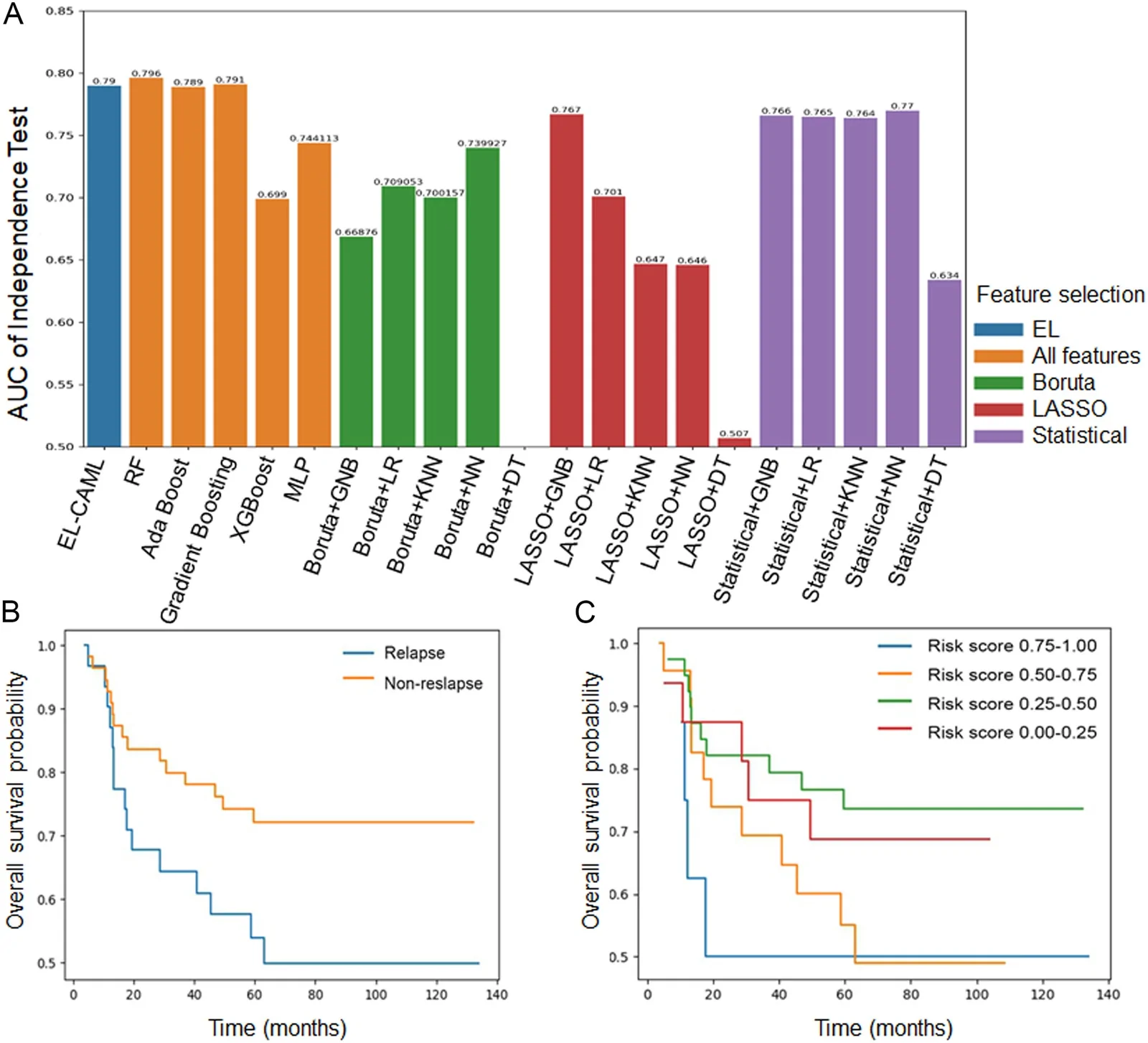
Predictive performance and prognostic stratification of the EL-CAML model. (A) Comparative performance of EL-CAML against baseline feature selection methods (Boruta, LASSO, and univariate statistical ranking) paired with multiple machine learning classifiers for pediatric AML relapse prediction. (B) Kaplan–Meier curves of overall survival for higher- and lower-risk strata defined using the rescaled SVM decision boundary of 0.5 (log-rank *P *= 0.045). The value 0.5 represents the model decision boundary and should not be interpreted as a universal clinical probability threshold. (C) Kaplan–Meier curves of overall survival across four intervals of the EL-CAML continuous relapse-risk score. Abbreviations: ML, machine learning; XGBoost, eXtreme Gradient Boosting; GNB, Gaussian naive Bayes; LR, logistic regression; KNN, k-nearest neighbors; DT, decision tree.

This parsimony signature showed less apparent overfitting than full-feature models, particularly in maintaining threshold-independent discriminatory performance in the independent evaluation cohort. As shown in Fig. 2A, full-feature ML models (e.g. MLP and RF) achieved higher training accuracy, but showed larger performance declines in validation settings, whereas EL-CAML maintained more stable score-based discrimination. This pattern is consistent with the high-dimensional, small-sample setting, in which accuracy-optimized models may capture cohort-specific variation. Furthermore, a comparison with the LASSO and Boruta feature selection methods indicated that EL-CAML identified a parsimony signature with improved out-of-sample predictive performance.

The EL-CAML relapse model generated continuous relapse-risk scores that stratified pediatric patient outcomes. KM survival analysis showed that patients assigned to the model-predicted relapse group based on a rescaled risk-score cutoff of 0.5 had significantly lower overall survival than those assigned to the model-predicted non-relapse group (log-rank *P *= 0.045; Fig. 2B). Score-stratified KM curves further illustrated overall survival trajectories across four EL-CAML relapse-risk score intervals (Fig. 2C). This survival pattern was consistent with the time-to-event concordance analysis (Table S3, available as supplementary data at *Bioinformatics* online), while the overall results support the interpretation of EL-CAML as a continuous relapse-risk ranking model. Regarding their reported roles in oncogenic regulation, the 18-miRNA signature included miRNAs previously annotated as tumor-suppressor miRNAs (tsmiRs) or oncogenic miRNAs (oncomiRs), as summarized in Table 1. Although several signature miRNAs showed disease-associated expression patterns and survival associations in independent analyses (Supplementary Figs. S5–S7, available as supplementary data at *Bioinformatics* online), univariate analysis indicated that no single miRNA was sufficient to predict relapse (Fig. S8, available as supplementary data at *Bioinformatics* online), supporting the need for a multivariate combinatorial signature.

### 3.2 Robustness and exploratory biological support of stability-aware miRNA anchors

Beyond the 18-miRNA parsimony signature, EL-CAML evaluated feature-level stability across 30 independent EL runs to identify complementary high-frequency miRNA features for downstream network construction. Using a selection-frequency threshold of *f_i_* ≥ 0.30, the complementary layer identified 10 complementary high-frequency miRNAs (*S_comp_*): miR-15b, miR-26a, miR-190a, miR-744, let-7f-2, miR-582, miR-3940, miR-3127, miR-3200, and miR-4741 (Supplementary Fig. S9, available as supplementary data at *Bioinformatics* online). These complementary biomarkers exhibited statistically significant biological enrichment relative to background expectation (*P *< 0.001), maintaining a mean validation AUC of 0.865 ± 0.017 and a mean test AUC of 0.811 ± 0.011. Consequently, TargetPrior integrated the 18-miRNA predictive signature (*S_cons_*) and the 10-miRNA complementary biomarkers (*S_comp_*) to establish a unified topological anchor set (*S_total_*, *F *= 28) for downstream network reconstruction.

In the co-expression-corrected robustness analysis, the high-frequency miRNA feature sets showed consistent recovery across repeated data perturbations (Supplementary Note 7, available as supplementary data at *Bioinformatics* online), with an overlap coefficient of 0.685 ± 0.147, Jaccard index of 0.441 ± 0.131, and Kuncheva index of 0.583 ± 0.135. These results support the computational reproducibility of the selected miRNA signals under sample-level perturbation, while remaining consistent with the feature-substitution behavior expected in high-dimensional transcriptomic data.

Because algorithmic stability does not necessarily imply biological relevance, we further examined the EL-derived miRNA anchors in the independent GSE196886 dataset. This dataset was used strictly as an exploratory biological support dataset, not as an external prognostic validation cohort. In the leukemic stem cell (LSC) versus normal hematopoietic stem cell (HSC) comparison, selected miRNAs showed compartment-associated expression differences: miR-27a and miR-3607 were upregulated, whereas miR-100 and miR-99b were downregulated in LSCs relative to HSCs (Fig. S10, available as supplementary data at *Bioinformatics* online). These results provide exploratory cellular-level support that a subset of TargetPrior-selected miRNAs is associated with AML progression-related stem-cell compartment dysregulation.

### 3.3 Literature-curated, DEG-constrained network and functional enrichment

**Figure 3:**
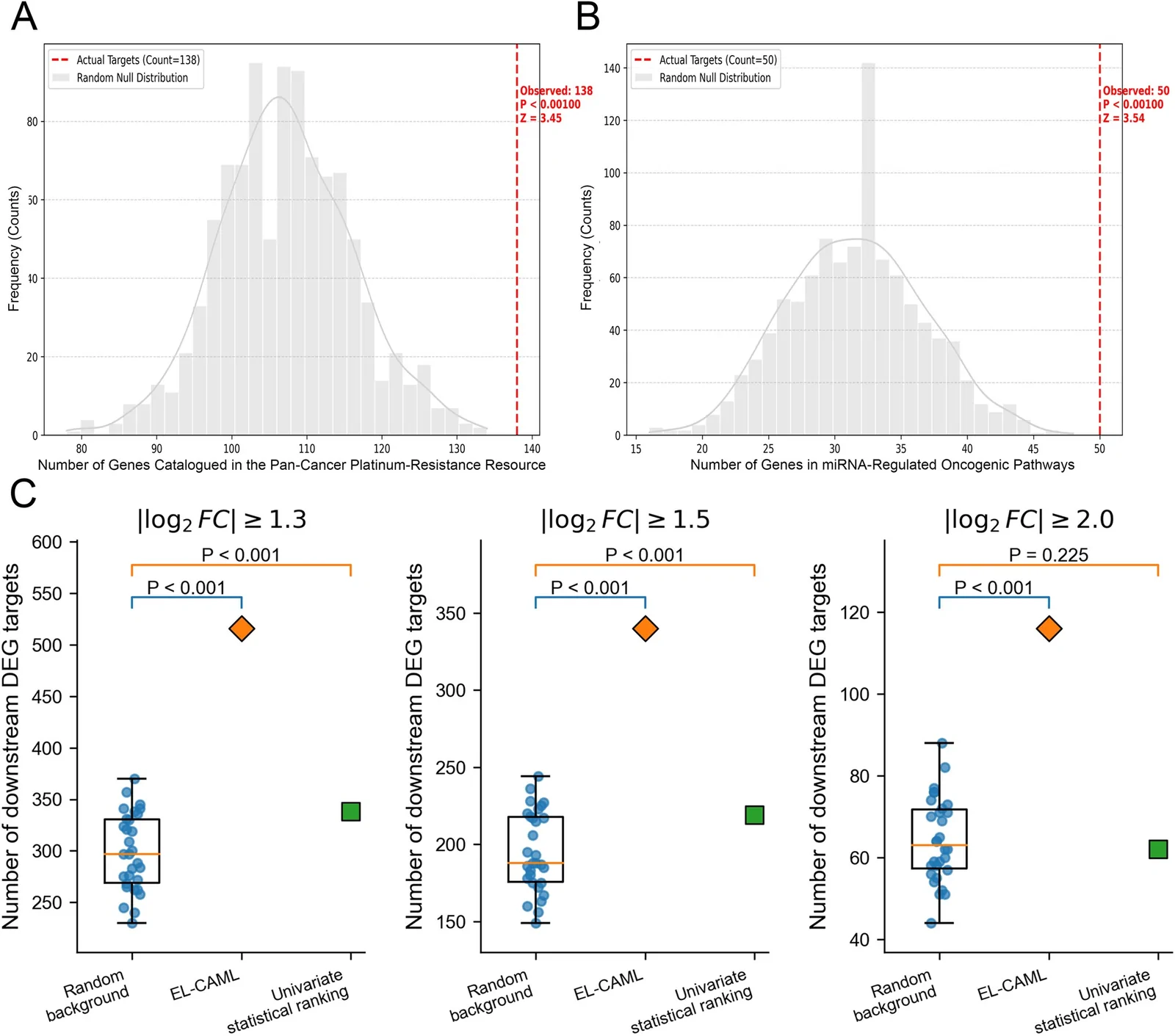
Topological centrality and permutation-based enrichment analysis of the TargetPrior regulatory network. (A, B) Permutation-based assessment of functional enrichment using Monte Carlo permutation testing (1000 iterations). Histograms display the null distributions of the numbers of target genes (A) catalogued in the pan-cancer platinum-resistance resource and (B) mapped to miRNA-regulated oncogenic pathways, generated by random sampling from the background miRNA pool. Red dashed lines indicate the observed target counts associated with the TargetPrior-derived topological anchors (*S_total_*). (C) Comparison of downstream DEG-target counts obtained using the EL-CAML anchor set, univariate statistical ranking, and size-matched random background controls at |log_2_FC| thresholds of 1.3, 1.5, and 2.0. The random background comprised 30 independently sampled sets of 28 miRNAs drawn without replacement from the 471 candidate miRNAs. Each random set was processed using the same miRTarBase interaction mapping and DEG-filtering procedure, and each blue point represents the number of unique downstream DEG targets obtained from one random set. The boxplots summarize these 30 background values, with the center line indicating the median, the box indicating the interquartile range, and the whiskers extending to 1.5 times the interquartile range. The orange diamonds and green squares indicate the single observed counts obtained using EL-CAML and univariate statistical ranking, respectively. *P*-values were calculated using two-sided one-sample *t*-tests comparing the random-background distribution with each observed strategy-specific target count (*P ≤ 0.05, ** *P *≤ 0.01, and *** *P *≤ 0.001). The mean numbers (± standard deviation [*SD*]) of background target genes at |log_2_FC| thresholds of 1.3, 1.5, and 2.0 were 298.80 ± 37.45, 195.20 ± 25.95, and 64.33 ± 10.31, respectively.

To characterize the biomolecular mechanisms of the identified topological anchors, we constructed a regulatory network by applying the stringent filtering thresholds (|log_2_ FC| ≥*τ_fc_* and adjusted *P*-value ≤ 0.05). These criteria reduced marginal low-effect transcriptomic variation and mapped the 28 miRNAs to 516 DEG-supported candidate target genes connected through miRTarBase-curated miRNA-target interactions. To rigorously verify that the observed network connectivity reflected specific biological functions rather than random artifacts, we conducted Monte Carlo permutation testing with 1000 iterations. The TargetPrior anchor set was enriched for genes catalogued in the pan-cancer PR resource and for miRNA-mediated oncogenic regulatory functions relative to the corresponding null distributions (*P *< 0.001; Fig. 3A and B).

Topological analysis further indicated that the EL-CAML anchor set yielded more downstream DEG targets than the 30 size-matched random background sets across increasing fold-change thresholds (|log_2_FC| ≥ 1.3, 1.5, and 2.0; two-sided one-sample t-test, all *P *< 0.001; Fig. 3C). The univariate statistical ranking strategy also yielded higher target counts than the random background at |log_2_FC| ≥ 1.3 and 1.5 (both *P *< 0.001), but not at |log_2_FC| ≥ 2.0 (*P *= 0.225). Together, these results indicate that stability-aware miRNA anchors prioritize DEG-supported, network-central candidate genes within the constrained regulatory network.

**Figure 4:**
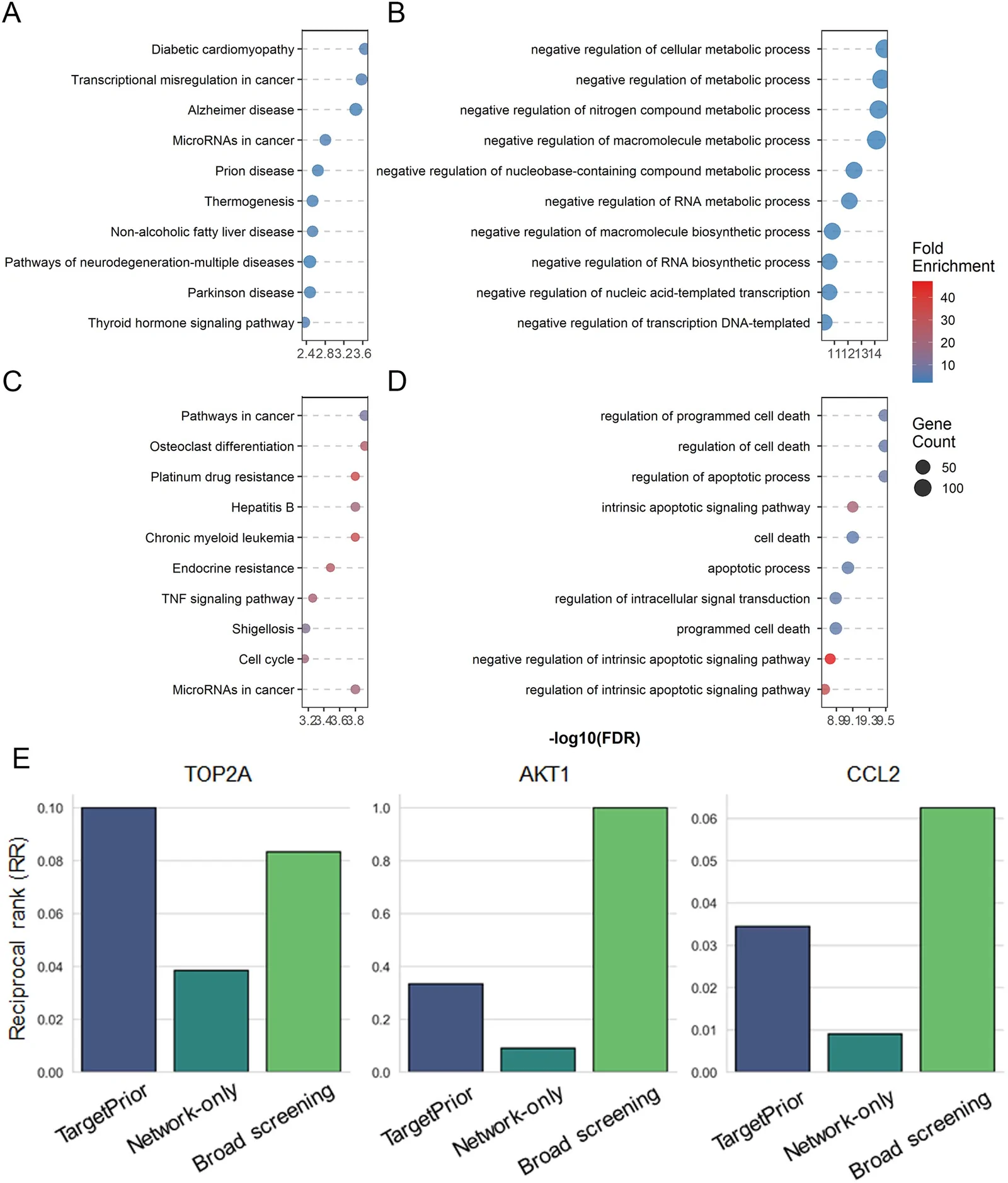
Functional enrichment of the regulatory network and benchmarking of target prioritization. (A–D) KEGG pathway and GO biological process enrichment analyses for (A, B) CAML relapse-associated network genes and (C, D) network genes catalogued in a pan-cancer platinum-resistance resource. Node size indicates gene count; the color gradient reflects the adjusted *P*-value. (E) Quantitative benchmarking of TargetPrior against baseline candidate target-prioritization strategies. Evaluation is based on the reciprocal rank (RR) of TARGET-cohort-relevant AML target references (*TOP2A*, *AKT1*, *CCL2*) using network closeness centrality as the primary ranking metric; additional centrality metrics were evaluated as sensitivity analyses in Table S2, available as supplementary data at *Bioinformatics* online.

Functional enrichment analyses of the *S_total_*-anchored regulatory network were conducted using two complementary gene sets. Analysis of CAML relapse-associated network genes identified cancer-related signaling and metabolic processes (Fig. 4A and B), whereas exploratory analysis of network genes catalogued in the pan-cancer PR resource identified pathways related to apoptosis, cancer-associated signaling, and metabolic regulation (Fig. 4C and D). Categories related to viral infection and neurological disorders also reached statistical significance (Supplementary Tables S4–S7, available as supplementary data at *Bioinformatics* online), which may reflect conserved cellular stress-response and basal metabolic programs. Among the 18-miRNA parsimony signature, 12 miRNAs were linked through curated miRNA–target interactions to genes catalogued in the pan-cancer PR resource, providing exploratory functional context for resistance-associated processes (Table S8, available as supplementary data at *Bioinformatics* online).

At the network annotation level, cross-cancer resistance-resource annotations are detailed in Table 2 and Table S9, available as supplementary data at *Bioinformatics* online. Top-ranked miRNAs, such as miR-27a and miR-106b, were linked through literature-curated post-transcriptional regulatory relationships to target transcripts of genes involved in signaling and apoptosis (e.g. *AKT1*, *TPT1*, *E2F1*). Beyond these specific interactions, the *S_cons_*-associated regulatory network contained genes annotated to DNA damage response, cancer stem cell maintenance, glycosylation, drug transport and efflux, and extracellular matrix organization. Collectively, these results provide network-level functional context for the prioritized candidate genes and support their use as therapeutic hypotheses for downstream validation, rather than as mechanistically validated drivers of CAML relapse.

**Table 2:** Exploratory annotation of genes catalogued in a pan-cancer platinum-resistance resource and linked to the ‘parsimony signature’ (*S_cons_*) within the cohort-constrained regulatory network.

Genes in the pan-cancer PR resource	Regulatory miRNAs (Scons)	Annotated functional processes	Representative enriched pathways
AKT1, UGCG, TPT1	miR-27a	Oncogenic, apoptotic, ceramide/efflux, Notch	AMPK, mTOR, p53, WNT
E2F1, MCL1, MDM2	miR-106b	Cell cycle, apoptosis, stress response	miRNAs in cancer
CCL2, DUSP1	let-7g	MAPK, immune response	MAPK signaling pathway
BCL2L2, MUC1, PML (broad coverage)	miR-335	Apoptosis, EMT, survival signaling, immune & metabolic regulation	miRNAs in cancer
MCL1	miR-133a-1	Apoptosis, Autophagy, EMT, ER stress	miRNAs in cancer
TOP2A, MCL1	miR-186	DDR, lipid metabolism, apoptosis	Mitochondrial energy metabolism, Oxidative stress–related metabolic injury, neurodegeneration-associated pathways, Cardiac muscle contraction
TFAM, E2F1	miR-1343	Cell cycle, HR repair, mitochondrial pathways	Cell cycle, Mitochondrial pathways
MEN1	miR-589	AKT signaling	PI3K-Akt signaling pathway
HSPA1B	miR-937	Autophagy, ER stress	Protein processing in endoplasmic reticulum
NACC1	miR-6842	EMT, autophagy	Autophagy, Pathways in cancer
ACTN4	miR-1538	ECM/EMT, WNT	Wnt signaling pathway, Focal adhesion
AKT1, TPT1	miR-100	AMPK/mTOR, redox, senescence	Cardiometabolic and hormonal signaling pathways, cAMP/cGMP-mediated second messenger signaling, Oxidative stress–related metabolic dysfunction, Neurodegeneration and cellular senescence pathways

Only miRNAs linked to at least one catalogued gene are displayed. The complete annotation of the 18-miRNA signature is available in Table S9, available as supplementary data at *Bioinformatics* online. Abbreviations: PR, platinum resistance; EMT, epithelial-mesenchymal transition; DDR, DNA damage response.

### 3.4 Benchmarking TargetPrior for drug target prioritization

Reducing the search space to experimentally testable therapeutic candidates is a pivotal challenge in translational bioinformatics. TargetPrior addresses this challenge by employing the EL-derived topological anchors (*S_total_*) to constrain the downstream regulatory search space. We benchmarked TargetPrior against four alternative strategies: a network-only baseline without miRNA-guided constraints; two size-matched feature selection strategies based on univariate statistics and LASSO regularization; and a broad screening strategy (*F_broad_*). The evaluated benchmark included three TARGET-cohort-relevant core references (*AKT1*, *TOP2A*, and *CCL2*) and six additional AML-associated target references (*RARA*, *FLT3*, *BCL2L2*, *MEN1*, *MCL1*, and *MDM2*) spanning clinically established, pharmacologically investigated, and biologically supported evidence tiers. These benchmark genes provided a curated pharmacological reference set for evaluating whether TargetPrior enriched AML-associated targets in the early ranking tiers.

As shown in Fig. 4E and Table S2, available as supplementary data at *Bioinformatics* online, TargetPrior achieved stronger early-rank retrieval of AML-associated target references than the network-only and statistical feature-selection baselines. For the three TARGET-cohort-relevant core references, TargetPrior achieved reciprocal ranks (RRs) of 0.333 for *AKT1*, 0.100 for *TOP2A*, and 0.034 for *CCL2*, substantially outperforming the unconstrained network-only baseline (*RR*: 0.091, 0.039, and 0.009, respectively). Using the expanded 9-gene AML reference set and the bias-controlled ranking evaluation described in Supplementary Note 7, available as supplementary data at *Bioinformatics* online, TargetPrior retrieved 8 of the 9 reference targets within the top 10 ranks of the constructed network, corresponding to Recall@10 = 0.889. Because eight reference targets were retrieved among the top 10 ranked candidates, Precision@10 = 0.80. Retrieval remained stable across broader thresholds, with Recall@K = 0.889 for *K* = 20, 50, and 100. The mean RR was 0.302, and the mean rank of retrieved reference targets was 53.778. Using 1000 degree-quantile-matched permutations to account for literature-driven degree bias, TargetPrior retained significant enrichment beyond the corresponding random expectation, with a permutation *P*-value < 0.001. Together, these results support the utility of TargetPrior as a topology-guided candidate prioritization framework that enriches AML-associated target references in the early ranking tiers and generates network-supported therapeutic hypotheses for downstream validation.

### 3.5 Prioritization of network-central candidate targets in therapeutically relevant functional classes

Extending the scope of target prioritization beyond curated AML-associated target references, TargetPrior identified network-central candidate genes spanning functional categories that include GTPases, phosphatases, epigenetic regulators, TFs, and genes overlapping the pan-cancer PR resource (Table 3; Supplementary Tables S10–S16, available as supplementary data at *Bioinformatics* online). Overall, 45 candidate genes were prioritized within the constrained regulatory network.

**Table 3:** Prioritization of 45 network-central candidate genes in therapeutically relevant functional classes by TargetPrior.

Functional target class	Prioritized candidate genes	Upstream regulatory miRNAs	Associated pharmacological agents
GTPases	RAP2B, GIMAP7, ARL8A, RHOB	miR-335, miR-190a, miR-589, miR-5642, miR-1343, miR-186, miR-6842	Botulinum toxin type A
Phosphatases	PPP3R1, PPP1CB, MPRIP, SSH3, INPPL1, CTDNEP1	miR-106b, miR-3127, miR-15b, miR-99b, miR-100, miR-27a, miR-335, miR-744	Myristic acid, Voclosporin
Epigenetics	TOP2A, DDIT4, PDRG1, DOT1L, DNAJC3, ID2, SMARCD1, CRAMP1, BICRA, BCL11B	miR-186, miR-335, miR-3607, miR-6874, miR-106b, miR-589, miR-6842, miR-744, miR-1271, miR-937, miR-3127, miR-582	TOP2A large drug list (anthracyclines, topoisomerase inhibitors, fluoroquinolones, etc.)
TFs	TFE3, ZNF777, USF2, ZNF592, ZBTB7B, MTA2, ZMIZ2, MAFF, TRERF1, RARA, KHSRP, NCOR2	miR-335, miR-106b, miR-1343, miR-6874, miR-937, miR-186, miR-27a, miR-744, miR-4741, miR-1538, miR-190a, miR-6842, miR-100, miR-15b	Adapalene, Acitretin, Alitretinoin, Tretinoin, Tazarotene, Etretinate, Isotretinoin, Tamibarotene, Trifarotene Resveratrol, Artenimol
PR-Resource	TGFB1, UGCG, SCRIB, YWHAH, BIRC5, MUC1, AKT1S1, NOTCH1, E2F1, BCL2L2, MCL1, CSF1, HSPA1B	miR-744, miR-15b, miR-335, miR-27a, miR-106b, miR-186, miR-26a, miR-3940, miR-4741, miR-1343, miR-582, miR-133a, miR-3127, miR-937, miR-100, miR-106b, miR-15b	Hyaluronidase, Terazosin, Miglustat, Eliglustat, Phenethyl isothiocyanate, Reserpine, Berberine

Candidates were identified and ranked based on network centrality metrics within the *S_total_*-anchored network, highlighting their roles as candidate targets for downstream evaluation. Abbreviations: TFs, transcription factors; PR, platinum resistance.

Topological analysis within the *S_total_*-anchored network revealed class-specific topological architectures across these functional classes. For instance, whereas genes encoding GTPases (e.g. *RAP2B*) generally occupied restricted, modular topological niches, prioritized phosphatases (e.g. *PPP3R1*, *MPRIP*) and epigenetic regulators (e.g. *TOP2A*, *DOT1L*) displayed high network centrality. The recovery of highly connected AML-relevant genes, including *TOP2A* and *AKT1*, provided internal consistency with established pharmacological references. Specifically, among all candidates, *AKT1* exhibited the highest connectivity and was annotated in relation to established antileukemic agents, including arsenic trioxide. Within the transcription-factor class, high-connectivity TFs included *TFE3* and *RARA*, with the latter being a well-established target for retinoid differentiation therapy. Collectively, these findings suggest that TargetPrior links transcriptomic miRNA anchors to network-prioritized candidate target genes for downstream experimental evaluation).

## 4 Discussion and conclusion

High-dimensional transcriptomic target prioritization remains challenging because many statistically comparable feature subsets can produce similar predictive performance. TargetPrior addresses this challenge by combining EL feature selection with stability-aware consensus aggregation. Rather than relying on a single optimized model, TargetPrior’s EL-driven search partly parallels the maximum relevance, minimum redundancy (mRMR) principle. This strategy may reduce the repeated selection of highly correlated variables, which tend to fractionate selection frequencies across iterations, thereby favoring features relevant to relapse status while limiting inter-feature redundancy. Consistent with our downstream functional analyses, this reduction in redundant feature co-selection may allow the parsimony signature to encompass distinct pathophysiological programs (e.g. metabolic reprogramming, apoptosis, and the DNA damage response) without extensive functional redundancy. Consequently, this functional complementarity supports broader network-level coverage within the *S_total_* network architecture.

Clinically, relapse-related AML mortality is strongly associated with chemoresistance mechanisms, such as leukemic stem cell dormancy and miRNA-mediated epigenetic reprogramming (Gibson *et al*. 2005, Li 2021, Jaaks *et al.* 2022, Niu *et al*. 2022), that can enable leukemic clones to evade frontline induction regimens. Because platinum resistance is not an established clinical resistance phenotype in AML, the Huang *et al.* resource was used only as a pan-cancer functional annotation layer. For CAML, TargetPrior provides a structured route from continuous relapse-risk scoring to therapeutic hypothesis generation. Recent network-based precision medicine approaches, such as NetAML, have integrated AML transcriptomic profiles with *ex vivo* drug-sensitivity data to support drug-specific response prediction (Wang 2025). Such approaches could provide a complementary downstream strategy for evaluating treatment-specific response patterns associated with TargetPrior-prioritized candidates. While conventional ML approaches identify prognostic biomarkers, they generally lack a systematic framework to translate these isolated prognostic features into candidate therapeutic targets (Lim *et al.* 2017, Wang *et al.* 2019, Radakovich *et al*. 2020, Wang 2022, Cao *et al*. 2023, Esperanza-Cebollada 2023, Kulaeva and Mashkina 2023). TargetPrior addresses this gap by using stability-selected miRNA anchors to construct and constrain a literature-curated, DEG-constrained regulatory network. This constraint reduces the search space from genome-wide transcriptomic variation to a focused set of network-linked candidate genes, thereby improving the interpretability of downstream target ranking. Intersecting the DEG pool with miRTarBase-curated targets of the 28 miRNA anchors yielded 516 DEG-supported candidate genes. The pharmacological benchmarking further supports stronger early-rank enrichment of AML-associated target references relative to network-only and statistical feature-selection baselines.

Rather than optimizing exclusively for in-sample predictive performance, EL-CAML emphasizes stability-aware and interpretable feature selection to support cross-cohort risk ranking and downstream target prioritization. It is important, however, to distinguish algorithmic stability from biological relevance. High selection stability does not establish biological causality or functional necessity, because technical artifacts, batch effects, or platform-specific biases may also be reproducibly selected. Within TargetPrior, stability-aware selection is therefore used as a computational reproducibility filter to reduce stochastic feature-selection variation and generate candidate miRNA anchors. The biological plausibility of these anchors was evaluated using complementary analyses, including compartment-associated dysregulation in the independent GSE196886 cell-sorted dataset, pathway enrichment beyond random expectation, and pharmacological benchmarking against AML-associated target references. These analyses support the prioritization utility and biological plausibility of the selected anchors, but they do not establish causal involvement, functional necessity, or clinical readiness. The external predictive evaluation also requires cautious interpretation.

The independent CCG2961 cohort was therefore interpreted as a small proof-of-concept evaluation cohort for continuous relapse-risk scoring, not as definitive external clinical validation. Although EL-CAML maintained threshold-independent discriminatory performance in the independent proof-of-concept cohort, the small cohort size (*n = *20) limits definitive conclusions regarding broad cross-cohort generalizability. The recalibration and threshold-sensitivity analyses further support interpreting EL-CAML as a continuous relapse-risk ranking model, not as a fixed-threshold classifier. Cross-cohort differences in expression platforms and cohort composition may shift absolute score distributions and attenuate fixed-threshold metrics, whereas ordinal risk information may remain partially preserved. Accordingly, any operational cutoff would require cohort-specific calibration and prospective validation before clinical deployment. Moreover, GSE196886 provides exploratory cellular-level support for selected miRNAs in AML-relevant cell-sorted populations, but the absence of relapse, survival, and time-to-event annotations precludes its use as an external prognostic validation cohort. This interpretation is consistent with the broader objective of TargetPrior, in which prediction is used to derive stability-selected miRNA anchors and target prioritization is performed through topology-guided ranking.

Although TargetPrior was developed and evaluated in CAML, its computational structure is adaptable across disease contexts. As a proof-of-concept extension (Supplementary Note 8, available as supplementary data at *Bioinformatics* online), the framework requires high-dimensional transcriptomic profiles, a phenotype of interest, and curated regulatory or drug-target knowledge bases. Supplementary cross-disease examples illustrate how analogous workflows could be configured for other pediatric cancer datasets, such as neuroblastoma or osteosarcoma. These examples should be regarded as conceptual extensions rather than empirical pan-cancer validation. Therefore, broader applicability must be tested in disease-specific cohorts with matched benchmarking resources and independent biological support or validation.

As a computational proof-of-concept, a primary limitation of this study is the requirement for independent experimental validation to establish causal regulatory roles and functional necessity. Furthermore, reliance on curated interaction databases inherently introduces literature bias, whereby extensively annotated genes (e.g. *AKT1*) may disproportionately accumulate interaction edges compared to underexplored, less-characterized targets. While the prioritized candidates, spanning from global hubs like *AKT1* to modular bottlenecks like *RAP2B*, provide testable regulatory hypotheses, their specific functional contributions within the dysregulated network warrant rigorous *in vitro* and *in vivo* assessment. Computational integration of functional genetic screening data, including combined dependency scores derived from CRISPR-Cas9 and shRNA screens, could provide complementary evidence for prioritizing candidates for experimental validation (Wang *et al.* 2019). Future studies employing targeted gene-editing strategies, such as CRISPR-Cas9 knockout screens, are needed to assess the functional relevance and necessity of these prioritized candidates in relevant AML models (Wang *et al*. 2021). Ultimately, targeting these network-derived candidate vulnerabilities in conjunction with conventional chemotherapeutic or antibody-based treatments may provide a testable strategy to investigate drug resistance (Yagishita *et al*. 2015, Huang *et al.* 2022, Rout 2022). Integrating these network-derived insights with systematic preclinical screening will be important for translating TargetPrior’s prioritized candidates into testable combinatorial therapies for CAML resistance management.

In conclusion, TargetPrior provides an interpretable computational framework that synergizes EL-based continuous relapse-risk scoring with topology-guided candidate target prioritization. The framework combines EL-based global optimization, stability-aware miRNA anchor selection, and literature-curated network topology to reduce the instability of high-dimensional biomarker selection and to prioritize AML-relevant candidate target genes. Rather than providing a universally transferable target-discovery rule, TargetPrior generates ranked therapeutic hypotheses that require disease-specific calibration, pharmacological benchmarking, and orthogonal experimental validation before clinical or mechanistic interpretation. This positioning supports its use as a structured bioinformatics framework for prioritizing candidate targets from transcriptomic datasets while maintaining appropriate caution regarding clinical translation and causal inference.

## Supplementary Material

btag635_Supplementary_Data

## Data Availability

All data, code, and ranked candidate lists are available on GitHub at https://github.com/NYCU-ICLAB/TargetPrior and have been archived on Zenodo (DOI: 10.5281/zenodo.20394263).

## References

[btag635-B1] Alam MS , Sultana A, Sun H et al Bioinformatics and network-based screening and discovery of potential molecular targets and small molecular drugs for breast cancer. Front Pharmacol 2022;13:942126. 36204232 10.3389/fphar.2022.942126 PMC9531711

[btag635-B2] Blair HA. Sotorasib: first approval. Drugs 2021;81:1573–9. 34357500 10.1007/s40265-021-01574-2 PMC8531079

[btag635-B3] Breiman L. Statistical modeling: the two cultures. Stat Sci 2001;16:199–215.

[btag635-B3311436] Cao C, , Wang T, , Luo Y et al Comprehensive analysis of cuproptosis-associated LncRNAs predictive value and related CeRNA network in Acute myeloid leukemia. Heliyon 2023;9:e22532. 10.1016/j.heliyon.2023.e22532 38058427 PMC10696213

[btag635-B4] Clarke R , Ressom HW, Wang A et al The properties of high-dimensional data spaces: implications for exploring gene and protein expression data. Nat Rev Cancer 2008;8:37–49. 18097463 10.1038/nrc2294 PMC2238676

[btag635-B5] Craig O , Lee S, Pilcher C et al A new method for network bioinformatics identifies novel drug targets for mucinous ovarian carcinoma. NAR Genom Bioinform 2024;6:lqae096. 39184376 10.1093/nargab/lqae096 PMC11344246

[btag635-B6] Crooke ST , Witztum JL, Bennett CF et al RNA-targeted therapeutics. Cell Metab 2018;27:714–39. 29617640 10.1016/j.cmet.2018.03.004

[btag635-B7] Dang CV , Reddy EP, Shokat KM et al Drugging the 'undruggable’ cancer targets. Nat Rev Cancer 2017;17:502–8. 28643779 10.1038/nrc.2017.36 PMC5945194

[btag635-B8] Darrason M. Mechanistic and topological explanations in medicine: the case of medical genetics and network medicine. Synthese 2018;195:147–73. 32214509 10.1007/s11229-015-0983-y PMC7089272

[btag635-B9] Donoho DL. Compressed sensing. IEEE Trans Inform Theory 2006;52:1289–306.

[btag635-B10] Ein-Dor L , Zuk O, Domany E. Thousands of samples are needed to generate a robust gene list for predicting outcome in cancer. Proc Natl Acad Sci U S A 2006;103:5923–8. 16585533 10.1073/pnas.0601231103 PMC1458674

[btag635-B2440256] Esperanza-Cebollada E, Gómez‐González S, Perez‐Jaume S et al A miRNA signature related to stemness identifies high-risk patients in paediatric acute myeloid leukaemia. Br J Haematol 2023;202:96–110. 36951259 10.1111/bjh.18746

[btag635-B11] Gibbs JB. Mechanism-based target identification and drug discovery in cancer research. Science 2000;287:1969–73. 10720316 10.1126/science.287.5460.1969

[btag635-B1896725] Gibson BES, , Wheatley K, , Hann IM et al Treatment strategy and long-term results in paediatric patients treated in consecutive UK AML trials. Leukemia 2005;19:2130–8. 10.1038/sj.leu.2403924 16304572

[btag635-B12] Gulezian E , Crivello C, Bednenko J et al Membrane protein production and formulation for drug discovery. Trends Pharmacol Sci 2021;42:657–74. 34270922 10.1016/j.tips.2021.05.006

[btag635-B13] Haibe-Kains B , El-Hachem N, Birkbak NJ et al Inconsistency in large pharmacogenomic studies. Nature 2013;504:389–93. 24284626 10.1038/nature12831 PMC4237165

[btag635-B14] Ho SY , Chen JH, Huang MH. Inheritable genetic algorithm for biobjective 0/1 combinatorial optimization problems and its applications. IEEE Trans Syst Man Cybern B Cybern 2004;34:609–20. 15369097 10.1109/tsmcb.2003.817090

[btag635-B15] Ho SY , Shu LS, Chen JH. Intelligent evolutionary algorithms for large parameter optimization problems. IEEE Trans Evol Computat 2004;8:522–41.

[btag635-B16] Hu H , Miao Y-R, Jia L-H et al AnimalTFDB 3.0: a comprehensive resource for annotation and prediction of animal transcription factors. Nucleic Acids Res 2019;47:D33–8. 30204897 10.1093/nar/gky822 PMC6323978

[btag635-B17] Huang D , Savage SR, Calinawan AP et al A highly annotated database of genes associated with platinum resistance in cancer. Oncogene 2021;40:6395–405. 34645978 10.1038/s41388-021-02055-2 PMC8602037

[btag635-B1889929] Huang L, , Zhang L, , Chen X. Updated review of advances in microRNAs and complex diseases: experimental results, databases, webservers and data fusion. Brief Bioinform 2022;23:bbac397. 10.1093/bib/bbac397 36094095

[btag635-B18] Ioannidis JP. Microarrays and molecular research: noise discovery? Lancet 2005;365:454–5. 15705441 10.1016/S0140-6736(05)17878-7

[btag635-B80163217] Jaaks P, , Coker EA, , Vis DJ et al Effective drug combinations in breast, colon and pancreatic cancer cells. Nature 2022;603:166–73. 10.1038/s41586-022-04437-2 35197630 PMC8891012

[btag635-B19] Jackstadt R , Hermeking H. MicroRNAs as regulators and mediators of c-MYC function. Biochim Biophys Acta 2015;1849:544–53. 24727092 10.1016/j.bbagrm.2014.04.003

[btag635-B19330308] Kulaeva ED, , Mashkina EV. Mrna-lncRNA geneexpression signature for predicting pediatric AML relapse. Curr Res Transl Med 2023;71:103379. 36738660 10.1016/j.retram.2023.103379

[btag635-B20] Kuron D , Pohlmann A, Angenendt L et al Amsacrine-based induction therapy in AML patients with cardiac comorbidities: a retrospective single-center analysis. Ann Hematol 2023;102:755–60. 36749402 10.1007/s00277-023-05111-x PMC9998561

[btag635-B0533217] Li Y, Wang Z, Ajani JA et al Drug resistance and Cancer stem cells. Cell Commun Signal 2021;19:19. 33588867 10.1186/s12964-020-00627-5 PMC7885480

[btag635-B97537] Lim EL, , Trinh DL, , Ries RE et al Microrna expression-based model indicates event-free survival in pediatric acute myeloid leukemia. J Clin Oncol 2017;35:3964–77. 10.1200/JCO.2017.74.7451 29068783 PMC5721230

[btag635-B21] Lin C-Y , Lee C-H, Chuang Y-H et al Membrane protein-regulated networks across human cancers. Nat Commun 2019;10:3131. 31311925 10.1038/s41467-019-10920-8 PMC6635409

[btag635-B22] Ma J , Hu J, Liu L et al Analysis of short-term efficacy and safety of mitoxantrone hydrochloride liposome regimen therapy in adult acute myeloid leukemia. JCO 2023;41:7039.

[btag635-B23] Menche J , Sharma A, Kitsak M et al Disease networks. Uncovering disease-disease relationships through the incomplete interactome. Science 2015;347:1257601. 25700523 10.1126/science.1257601 PMC4435741

[btag635-B24] Mohs RC , Greig NH. Drug discovery and development: role of basic biological research. Alzheimers Dement (N Y) 2017;3:651–7. 29255791 10.1016/j.trci.2017.10.005 PMC5725284

[btag635-B25] Morselli Gysi D , Do Valle Í, Zitnik M et al Network medicine framework for identifying drug-repurposing opportunities for COVID-19. Proc Natl Acad Sci U S A 2021;118:e2025581118. 33906951 10.1073/pnas.2025581118 PMC8126852

[btag635-B5148522] Radakovich N, , Cortese M, , Nazha A. Acute myeloid leukemia and artificial intelligence, algorithms and new scores. Best Pract Res Clin Haematol 2020;33:101192. 10.1016/j.beha.2020.101192 33038981 PMC7548395

[btag635-B4954918] Niu J, , Peng D, , Liu L. Drug resistance mechanisms of Acute Myeloid Leukemia stem cells. Front Oncol 2022;12:896426. 35865470 10.3389/fonc.2022.896426 PMC9294245

[btag635-B26] Raver-Shapira N , Marciano E, Meiri E et al Transcriptional activation of miR-34a contributes to p53-mediated apoptosis. Mol Cell 2007;26:731–43. 17540598 10.1016/j.molcel.2007.05.017

[btag635-B27] Röllig C , Steffen B, Schliemann C et al Single or double induction with 7 + 3 containing standard or high-dose daunorubicin for newly diagnosed AML: the randomized DaunoDouble trial by the study alliance leukemia. J Clin Oncol 2025;43:65–74. 39284116 10.1200/JCO.24.00235

[btag635-B8165225] Rout SR, Kenguva G, Singh V et al Chapter 12- combination of anticancer drugs with microrna as cancer therapeutics. In: Combination Drugdelivery Approach as an Effective Therapy for Various Diseases. Academic Press, 2022, 273–95.

[btag635-B28] Sathipati SY , Tsai M-J, Aimalla N et al An evolutionary learning-based method for identifying a circulating miRNA signature for breast cancer diagnosis prediction. NAR Genom Bioinform 2024;6:lqae022. 38406797 10.1093/nargab/lqae022 PMC10894035

[btag635-B29] Sherif HA , Magdy A, Elshesheni HA et al Treatment outcome of doxorubicin versus idarubicin in adult acute myeloid leukemia. Leuk Res Rep 2021;16:100272. 34692402 10.1016/j.lrr.2021.100272 PMC8517376

[btag635-B30] Tiwari PC , Pal R, Chaudhary MJ et al Artificial intelligence revolutionizing drug development: exploring opportunities and challenges. Drug Dev Res 2023;84:1652–63. 37712494 10.1002/ddr.22115

[btag635-B32512150] Wang Q, Yue C, Liu Q et al Explorationof differentially expressed mRNAs and miRNAs for pediatric acute myeloidleukemia. Front Genet 2022;13:865111. 36160019 10.3389/fgene.2022.865111 PMC9499657

[btag635-B9431209] Wang Y, Liu R, Zhang Y et al A network-driven framework for drug response precision prediction of Acute Myeloid Leukemia. Adv Sci (Weinh) 2025;12:e06447 40642825 10.1002/advs.202506447 PMC12463123

[btag635-B6435644] Wang P, , Zhou Y, , Richards AM. Effective tools for RNA-derived therapeutics: siRNA interference or miRNA mimicry. Theranostics 2021;11:8771–96. 10.7150/thno.62642 34522211 PMC8419061

[btag635-B4139208] Wang J-D, , Zhou H-S, , Tu X-X et al Prediction of competing endogenous RNA coexpression network as prognostic markers in AML. Aging (Albany NY) 2019;11:3333–47. 10.18632/aging.101985 31164492 PMC6555472

[btag635-B31] Wishart DS , Feunang YD, Guo AC et al DrugBank 5.0: a major update to the DrugBank database for 2018. Nucleic Acids Res 2018;46:D1074–82. 29126136 10.1093/nar/gkx1037 PMC5753335

[btag635-B32] Xie S , Liu H, Zhu S et al Arsenic trioxide and p97 inhibitor synergize against acute myeloid leukemia by targeting nascent polypeptides and activating the ZAKalpha-JNK pathway. Cancer Gene Ther 2024;31:1486–97. 39122830 10.1038/s41417-024-00818-z PMC11489083

[btag635-B33] Xie X , Yu T, Li X et al Recent advances in targeting the "undruggable" proteins: from drug discovery to clinical trials. Signal Transduct Target Ther 2023;8:335. 37669923 10.1038/s41392-023-01589-z PMC10480221

[btag635-B7227933] Yagishita S, , Fujita YU, , Kitazono S et al Chemotherapy-regulated microRNA-125-HER2 pathway as a novel therapeutic target for Trastuzumab-Mediated Cellular Cytotoxicity in Small Cell Lung Cancer. Mol Cancer Ther 2015;14:1414–23. 10.1158/1535-7163.MCT-14-0625 25833836

[btag635-B34] Yerukala Sathipati S , Aimalla N, Tsai M-J et al Prognostic microRNA signature for estimating survival in patients with hepatocellular carcinoma. Carcinogenesis 2023a;44:650–61. 37701974 10.1093/carcin/bgad062

[btag635-B35] Yerukala Sathipati S , Ho SY. Identifying the miRNA signature associated with survival time in patients with lung adenocarcinoma using miRNA expression profiles. Sci Rep 2017;7:7507. 28790336 10.1038/s41598-017-07739-y PMC5548864

[btag635-B36] Yerukala Sathipati S , Ho SY. Survival associated miRNA signature in patients with head and neck carcinomas. Heliyon 2023;9:e17218. 37360084 10.1016/j.heliyon.2023.e17218 PMC10285236

[btag635-B37] Yerukala Sathipati S et al Artificial intelligence-driven pan-cancer analysis reveals miRNA signatures for cancer stage prediction. HGG Adv 2023b;4:100190. 37124139 10.1016/j.xhgg.2023.100190 PMC10130501

[btag635-B38] You Y , Lai X, Pan Y et al Artificial intelligence in cancer target identification and drug discovery. Signal Transduct Target Ther 2022;7:156. 35538061 10.1038/s41392-022-00994-0 PMC9090746

[btag635-B39] Zhang S , Cheng Z, Wang Y et al The risks of miRNA therapeutics: in a drug target perspective. Drug Des Devel Ther 2021;15:721–33. 10.2147/DDDT.S288859 PMC7910153 33654378

[btag635-B40] Zhang W , Gou P, Dupret J-M et al Etoposide, an anticancer drug involved in therapy-related secondary leukemia: enzymes at play. Transl Oncol 2021;14:101169. 34243013 10.1016/j.tranon.2021.101169 PMC8273223

[btag635-B41] Zhang Y , Li X, Weng X et al Optimization of idarubicin and cytarabine induction regimen with homoharringtonine for newly diagnosed acute myeloid leukemia patients based on the peripheral blast clearance rate: a single-arm, phase 2 trial (RJ-AML 2014). Am J Hematol 2022;97:43–51. 34687467 10.1002/ajh.26386

[btag635-B42] Zou H , Hastie T. Regularization and variable selection via the elastic net. J R Stat Soc Ser B Stat Methodol 2005;67:301–20.

