# Supplementary material for: TargetPrior: a miRNA-signature embedded evolutionary learning framework for prioritizing drug targets in acute myeloid leukemia
Source: Bioinformatics. 2026 Aug 27;42(9):btag635. doi: 10.1093/bioinformatics/btag635 (PMC13553084; doi:10.1093/bioinformatics/btag635)
Supplement: Supplementary file 1 [file btag635_supplementary_data.zip › Supplementary.docx]

**Supplementary Note 1. Construction of an expanded pharmacological benchmark set for AML target prioritization.**

To evaluate the prioritization efficacy of the TargetPrior framework and support bias-aware pharmacological benchmarking, we established a curated AML target reference set comprising nine clinically established, pharmacologically investigated, or biologically supported AML-associated target references for acute myeloid leukemia (AML). These targets were curated from multiple evidence tiers, including FDA-approved AML drug labels, late-phase or mechanism-driven clinical investigations, and established AML-relevant pharmacological or biological mechanisms. To reflect the pathophysiological heterogeneity of AML, the benchmark set was organized into three core mechanistic categories:

The primary evaluation core comprises three targets with documented relevance to chemotherapy response or the leukemic microenvironment in context of the primary TARGET-AML cohort: (1) *AKT1* (AKT serine/threonine kinase 1), which has been implicated in leukemogenic signaling and drug-resistance-associated pathways. *AKT1* has a reported pharmacological association with arsenic trioxide (Xie, et al., 2024), which has shown efficacy in specific leukemia subtypes. (2) *TOP2A* (DNA topoisomerase II alpha), a primary target for a broad spectrum of anthracyclines and topoisomerase inhibitors, forming the backbone of AML induction therapy. Associated drugs include amsacrine (Kuron, et al., 2023), daunorubicin (Rollig, et al., 2025), etoposide (Zhang, et al., 2021), doxorubicin (Sherif, et al., 2021), idarubicin (Zhang, et al., 2022), mitoxantrone (Ma, et al., 2023). (3) *CCL2* (C-C motif chemokine ligand 2), a chemokine associated with the leukemia microenvironment and immune evasion. *CCL2* was included based on reported drug-related and microenvironmental relevance, including danazol-associated evidence (Colunga-Pedraza, et al., 2018), rather than as evidence of direct functional validation in this study.

To further support AML-relevant target-ranking evaluation, we expanded this benchmark with six additional AML-relevant reference genes spanning kinase signaling, differentiation therapy, epigenetic regulation, apoptosis, and p53 regulation: (4) *FLT3*, a well-established AML-associated therapeutic vulnerability targeted by FDA-approved FLT3-targeted inhibitors (midostaurin, gilteritinib, quizartinib) (Erba, et al., 2023; Perl, et al., 2019; Stone, et al., 2017); (5) *RARA*, included to represent the retinoic-acid differentiation axis in acute promyelocytic leukemia (APL), in which PML::RARA is a defining therapeutic lesion targeted by tretinoin and arsenic trioxide (Borate, et al., 2024); (6) *MEN1* (menin), an epigenetic regulatory node pharmacologically targeted by the FDA-approved menin inhibitor revumenib in relapsed/refractory leukemias harboring *KMT2A* translocations or susceptible *NPM1* mutations (Issa, et al., 2025); (7) *BCL2L2* (Bcl-w), an anti-apoptotic BCL-2 family member implicated in venetoclax resistance in AML and investigated in the context of broader BH3-mimetic strategies (e.g., navitoclax, AZD0466) (Shah, et al., 2026); (8) *MCL1*, an AML survival-associated anti-apoptotic protein and reported venetoclax-resistance mediator targeted by emerging selective inhibitors (e.g., S64315/MIK665, AZD5991) (Hu, et al., 2025); and (9) *MDM2*, a negative regulator of the p53 pathway investigated in AML clinical trials (e.g., idasanutlin) (Montesinos, et al., 2020).

Collectively, this expanded 9-gene reference set served two computational purposes. First, it enabled a quantitative assessment of target-reference retrieval across ranking metrics, including Precision@K, Recall@K, and mean reciprocal rank (MRR). Second, it supported degree-quantile-matched topological permutation testing to assess whether TargetPrior preferentially retrieved AML-associated target references beyond what would be expected from historically over-annotated or highly connected network hubs.

Borate, U.*, et al.* AML-347 SELECT-AML-1: Phase 2 Randomized Trial of Tamibarotene in Combination With Venetoclax and Azacitidine in Adult Patients With Previously Untreated AML With RARA Overexpression, Who Are Ineligible for Standard Induction Therapy. *Clinical Lymphoma Myeloma and Leukemia* 2024;24:S307.

Colunga-Pedraza, P.R.*, et al.* Danazol as First-Line Therapy for Myelodysplastic Syndrome. *Clin Lymphoma Myeloma Leuk* 2018;18(2):e109-e113.

Erba, H.P.*, et al.* Quizartinib plus chemotherapy in newly diagnosed patients with FLT3-internal-tandem-duplication-positive acute myeloid leukaemia (QuANTUM-First): a randomised, double-blind, placebo-controlled, phase 3 trial. *Lancet* 2023;401(10388):1571-1583.

Hu, X.*, et al.* Src inhibition potentiates MCL-1 antagonist activity in acute myeloid leukemia. *Signal Transduct Target Ther* 2025;10(1):50.

Issa, G.C.*, et al.* Menin Inhibition With Revumenib for KMT2A-Rearranged Relapsed or Refractory Acute Leukemia (AUGMENT-101). *J Clin Oncol* 2025;43(1):75-84.

Kuron, D.*, et al.* Amsacrine-based induction therapy in AML patients with cardiac comorbidities: a retrospective single-center analysis. *Ann Hematol* 2023;102(4):755-760.

Ma, J.*, et al.* Analysis of short-term efficacy and safety of mitoxantrone hydrochloride liposome regimen therapy in adult acute myeloid leukemia. *Journal of Clinical Oncology* 2023;41(16_suppl):7039-7039.

Montesinos, P.*, et al.* MIRROS: a randomized, placebo-controlled, Phase III trial of cytarabine +/- idasanutlin in relapsed or refractory acute myeloid leukemia. *Future Oncol* 2020;16(13):807-815.

Perl, A.E.*, et al.* Gilteritinib or Chemotherapy for Relapsed or Refractory FLT3-Mutated AML. *N Engl J Med* 2019;381(18):1728-1740.

Rollig, C.*, et al.* Single or Double Induction With 7 + 3 Containing Standard or High-Dose Daunorubicin for Newly Diagnosed AML: The Randomized DaunoDouble Trial by the Study Alliance Leukemia. *J Clin Oncol* 2025;43(1):65-74.

Shah, R.*, et al.* Evaluation of dual BCL-2/BCL-XL inhibition in acute myeloid leukemia preclinical models with and without prior venetoclax therapy. *Exp Hematol* 2026;156:105379.

Sherif, H.A.*, et al.* Treatment outcome of doxorubicin versus idarubicin in adult acute myeloid leukemia. *Leuk Res Rep* 2021;16:100272.

Stone, R.M.*, et al.* Midostaurin plus Chemotherapy for Acute Myeloid Leukemia with a FLT3 Mutation. *N Engl J Med* 2017;377(5):454-464.

Xie, S.*, et al.* Arsenic trioxide and p97 inhibitor synergize against acute myeloid leukemia by targeting nascent polypeptides and activating the ZAKalpha-JNK pathway. *Cancer Gene Ther* 2024;31(10):1486-1497.

Zhang, W.*, et al.* Etoposide, an anticancer drug involved in therapy-related secondary leukemia: Enzymes at play. *Transl Oncol* 2021;14(10):101169.

Zhang, Y.*, et al.* Optimization of idarubicin and cytarabine induction regimen with homoharringtonine for newly diagnosed acute myeloid leukemia patients based on the peripheral blast clearance rate: A single-arm, phase 2 trial (RJ-AML 2014). *Am J Hematol* 2022;97(1):43-51.

**Supplementary Note 2. Detailed processing pipeline for the independent cell-sorted pediatric AML small RNA-seq dataset (GSE196886).**

To assess compartment-specific expression support for the TargetPrior-derived signature, raw miRNA count data from an independent pediatric AML dataset (GEO: GSE196886) were retrieved, encompassing 18 sorted cellular subpopulation libraries.

The raw counts were converted to transcripts per million (TPM) by normalizing against mature miRNA sequence lengths extracted from the miRBase reference database. Non-zero values were subsequently log_2_-transformed (log_2_TPM) to reduce scale dispersion, while zero values were explicitly retained as zero. Lymphocyte subpopulations were excluded from downstream evaluation to reduce lineage confounding. Fold-change-based expression comparisons were performed using predefined cellular sorting metadata. For the primary leukemic stem cell (LSC) versus hematopoietic stem cell (HSC) comparison, the LSC compartment was represented by the sorted profiles of K16/2924, K16/2930, K16/2933, and K16/2936, which were compared against the normal HSC reference profiles K16/1322 and K16/2883. Features meeting the magnitude threshold of |log2FC| ≥ 1 were prioritized as compartment-associated miRNA expression changes, providing an independent exploratory biological support layer separate from the clinical predictive cohort.

**Supplementary Note 3. Detailed implementation of the EL-CAML algorithm.**

**Algorithmic framework**

The core feature selection engine of TargetPrior, EL-CAML (evolutionary learning for childhood acute myeloid leukemia), is built upon the inheritable bi-objective combinatorial genetic algorithm (IBCGA). The primary objective of EL-CAML is to formulate miRNA feature selection as a combinatorial optimization problem, in which a compact subset of discriminative miRNA features is selected from a high-dimensional feature space consisting of *p* features while maximizing cross-validated predictive performance. EL-CAML functions by executing multiple independent runs (*R* runs) of IBCGA incorporated with support vector machine (SVM) classifier to identify repeatedly selected candidate miRNA features.

**Encoding and intelligent evolutionary operations**

EL-CAML utilizes a binary chromosome encoding scheme to represent potential solutions. Each individual is represented by a binary chromosome of length *p*, in which *m* bits are set to 1 and the remaining *p* − *m* bits are set to 0. A value of 1 indicates that the corresponding miRNA feature is selected, whereas 0 indicates exclusion. To enable the simultaneous co-optimization of feature subsets and SVM hyperparameters, the binary chromosome is extended beyond the length of *p*. Specifically, additional binary segments of lengths *l_C_* and *l_γ_* are appended to the chromosome to encode the penalty parameter *C* and the radial basis function (RBF) kernel width γ, respectively. The total length of the chromosome becomes *p* + *l_C_* + *l_γ_*. During fitness evaluation, these appended binary substrings are decoded into real-valued hyperparameters mapped to a predefined logarithmic search space. The search ranges were set from 10⁻¹ to 10³ for *C*, and from 10⁻⁴ to 10¹ for *γ*. This extended encoding scheme allows candidate feature subsets and SVM hyperparameter configurations to be optimized jointly. To effectively explore the vast combinatorial space, IBCGA incorporates an orthogonal experimental design (OED)-based crossover operator, distinguishing it from traditional genetic algorithms that rely on random crossover. This ‘intelligent’ mechanism systematically samples potential combinations of feature indicators and hyperparameter-encoding segments from two parent chromosomes to identify the higher-fitness offspring, thereby enhancing search efficiency and convergence speed. The mutation operation is implemented as a swap operation (exchanging one selected bit '1' with one unselected bit '0') to maintain the fixed subset size *m* while preserving population diversity.

**Bi-objective fitness function and model optimization**

The fitness evaluation in EL-CAML is driven by a bi-objective function designed to balance predictive performance and model complexity. The algorithm seeks to simultaneously maximize the predictive performance and minimize the feature subset size (*m*). Predictive performance is quantified using the Matthews correlation coefficient derived from 5-fold cross-validation (5-CV) on the training set. We utilized an SVM classifier with an RBF kernel. Importantly, the evolutionary chromosome encodes not only the feature selection bits but also the SVM hyperparameters (the penalty parameter *C* and the RBF kernel width γ). This allows EL-CAML to co-optimize the feature subset and the model parameters within the same evolutionary loop, thereby reducing dependence on suboptimal fixed hyperparameter settings and allowing each selected subset to be evaluated under its jointly tuned model configuration.

**Step-by-step execution of IBCGA**

In a single execution, IBCGA operates through an inheritance mechanism that progressively reduces the current feature subset size *r* from a start point (*r_start_*) to an end point (*r_end_*). The default parameters used in this study were: *N_pop_* = 50, *r_start_* = 30, *r_end_* = 10, selection probability *P_s_* = 1.0, crossover probability *P_c_* = 0.8, mutation probability *P_m_* = 0.05, maximum generations *G_max_* = 1000, and convergence parameter *Convergence_max_* = 50. The main algorithm iterates through initialization, fitness evaluation, selection, OED-based crossover, and mutation. The convergence checking mechanism determines whether additional generations are required at the current subset size *r* before the inheritance operator reduces the subset size. This process continues until *r* reaches *r_end_*, outputting the best-performing signature for all scanned sizes.

Step 1 (Initialization): Randomly generate an initial population of *N_pop_* individuals. Set *r* = *r_start_* and *Gen* = 0.

Step 2 (Evaluation): Evaluate the fitness values of all *N_pop_* individuals using 5-CV SVM models.

Step 3 (Selection): Generate a new mating pool of *P_s_* × *N_pop_* winners using tournament selection (tournament size = 2), where the winner is selected from each pair of randomly chosen individuals.

Step 4 (OED-based crossover): Randomly select *P_c_* × *N_pop_* pairs of parents and perform an orthogonal experimental design-based crossover operation.

Step 5 (Mutation): Randomly select *P_m_* × *N_pop_* individuals to perform swap mutation. The current best individual is excluded from mutation through elitist preservation to preserve the current best solution.

Step 6 (Convergence checking): Increase *Gen* by one. When *r* = *r_start_*, the algorithm runs until *Gen* = *G_max_* to ensure that the initial high-dimensional subset is sufficiently optimized before progressive inheritance-based feature reduction. Because smaller subsets (*r* < *r*_start_) are derived from this solution, establishing a high-quality starting point is essential for maintaining predictive performance across decreasing signature sizes. For *r* < *r*_start_, the stopping condition is defined as either reaching *G_max_* generations or no improvement in fitness for *Convergence_max_* consecutive generations. If the stopping condition is satisfied, the best individual is recorded as the best-performing signature for the current size *r* and *Gen* is reset to 0; otherwise, return to Step 2.

Step 7 (Termination checking): If *r* = *r_end_*, stop the algorithm and output the feature subset with the highest fitness obtained among all fully evaluated feature subsets.

Step 8 (Inheritance): For each individual in the population, randomly select one active feature bit (bit value = 1) and change it to 0. Decrease *r* by one and go to Step 2.

**Stability-based consensus selection**

To address the Rashomon effect (model multiplicity) inherent in underdetermined problems, EL-CAML aggregates results from the *R* independent runs to finalize feature selection based on two distinct stability metrics:

A feature-level selection frequency(*f_i_*) is used to evaluate stability at the individual feature level. We calculate *f_i_* for each feature *i* as follows:

$$f_{i}=\frac{1}{R}\sum_{r=1}^{R} I_{r,i}$$

where $I_{r,i}$ is the indicator function that equals 1 if feature *i* is selected in the selected feature subset of the *r*-th run.

A subset-level stability score, denoted as *SSS(S)*, is used to evaluate subset-level reproducibility across independent runs. Rather than merely counting exact matches of entire subsets, this metric summarizes the collective selection reproducibility of a candidate subset *S* by averaging the selection frequencies of its constituent features:

$$SSS\left( S \right)=\frac{1}{m}\sum_{i\in S} f_{i}$$

where *m = |S|* represents the size of the feature subset. The subset with the highest subset-level stability score is identified as the ‘parsimony signature’ (denoted as *S_cons_*), representing the compact predictive subset with the highest subset-level stability score among candidate subsets.

Finally, features satisfying a defined stability threshold (*f_i_* ≥ *θ*) are designated as 'complementary biomarkers' (denoted as *S_comp_*). To ensure mutually exclusive sets (*S_cons_* $\cap$ *S_comp_* = $\emptyset$), features already present in *S_cons_* are strictly excluded. These *S_comp_* biomarkers are incorporated as additional miRNA anchors for downstream regulatory network construction. (Note: In our current CAML application, the specific parameters were *p* = 471, *R* = 30, |*S_cons_*| = 18 (*m* = 18), *θ* = 0.30, and |*S_comp_*| = 10.)

**Supplementary Note 4. Risk score normalization, recalibration, and survival analysis**

**Piecewise linear risk score normalization**

The EL-CAML support vector machine (SVM) classifiers outputs continuous decision values. To map these into a bounded [0, 1] interval for continuous risk stratification without disrupting the ordinal ranking, a monotonic piecewise linear normalization was applied. The mathematical decision boundary (value = 0) was anchored to 0.5, while the maximum positive and minimum negative decision values from the training cohort are used as scaling anchors for 1 and 0, respectively. Because this mapping is not fitted to empirical outcome probabilities, it functions purely as an uncalibrated ordinal risk score rather than a fully calibrated probability.

**Probability recalibration and threshold sensitivity**

To test whether score-distribution shifts could be mitigated, post hoc recalibrations (Platt scaling and Isotonic regression) were fitted on a training-derived calibration subset. Neither parametric nor non-parametric recalibration improved the Brier score or expected calibration error (ECE) on the independent test set, indicating that score-level recalibration alone was insufficient to improve calibration under cohort-level distributional shift in this setting.

**Survival-specific risk stratification**

To evaluate EL-CAML as a continuous risk stratification tool, time-to-event metrics were employed. Ordinal survival risk discrimination was quantified using Harrell’s concordance index (C-index), while time-specific prediction error was evaluated using the 36-month inverse probability of censoring weighting (IPCW) Brier Score. These metrics were used to assess whether the continuous score retained patient-level risk-ordering information independent of arbitrary binary thresholds.

Although relapse status is formulated as a binary target variable, EL-CAML is interpreted as a continuous relapse-risk scoring model rather than a universal fixed-threshold classifier. The SVM decision function produces a signed margin that reflects the relative position of each patient with respect to the learned relapse/non-relapse boundary. We therefore use the transformed score primarily to preserve ordinal relapse-risk information. Threshold-dependent metrics are reported for comparability with conventional binary prediction studies, whereas AUC and survival stratification analyses are used to evaluate threshold-independent discrimination and survival-associated risk separation. Any clinical decision threshold would require cohort-specific calibration and prospective validation before deployment.

**Supplementary Note 5. Baseline methods for robustness validation.**

To assess whether the miRNA sets identified by TargetPrior, including the parsimony signature [*S_cons_*] and the complementary biomarkers [*S_comp_*], exhibited network-level properties beyond size-matched random feature selection, we implemented two baselines for comparative assessment. The first baseline was a size-matched random selection control, designed to establish a null distribution for topological connectivity and DEG enrichment. In this procedure, we randomly selected *F* features from the global feature space (consisting of *p* features) without replacement, where *F* was explicitly defined as the total size of the unified TargetPrior-derived topological anchor set (*F* = *m* + *k*, comprising the *m*-feature core signature and *k* complementary biomarkers). This random sampling process was repeated for *N_rep_* independent iterations. Each sampled miRNA set was processed using the same miRTarBase interaction mapping and DEG-filtering procedure as Stotal. At each |log_2_FC| threshold of 1.3, 1.5, and 2.0, the number of unique downstream DEG targets obtained from one random set constituted an individual data point in the background boxplot in Fig. 3C.

The second baseline was a statistical filtering benchmark, which evaluated whether the multivariate, combinatorial search of EL-CAML provided additional prioritization value beyond standard univariate ranking. We ranked all *p* miRNAs based on the raw *P*-values derived from the Wilcoxon rank-sum test comparing clinical outcome groups (relapsed versus non-relapsed cases). The top *F* features with the smallest *P*-values were selected to form a univariate statistical signature. The downstream DEG-target count obtained from this single size-matched signature was displayed as the observed value for the univariate statistical-ranking strategy in Fig. 3C. By comparing the downstream regulatory targets of this univariate statistical signature against the EL-derived topological anchor set (*S_total_*) under the same network construction and evaluation pipeline, we evaluated whether EL-CAML captured multivariate feature patterns not represented by marginal univariate ranking. In the current CAML application, these parameters were set as *p* = 471, *m* = 18, *k* = 10, *F* = 28, and *N_rep_* = 30.

**Supplementary Note 6. Machine learning classifiers and hyperparameter settings.**

To benchmark the performance of the EL-CAML-derived ‘parsimony signature’ (*S_cons_)*, we compared it against a representative set of standard machine learning classifiers implemented using scikit-learn v1.1.2 and TensorFlow. The comparative analysis encompassed neural network architectures, ensemble learning strategies, and baseline classifiers. Consistent with the primary analysis, all models were evaluated using a unified 5-CV framework. Feature subset sizes for comparative feature selection methods (e.g., LASSO, Boruta) were controlled where applicable to align with the EL-CAML signature size (*m*), reducing confounding by feature-set size when comparing predictive performance. In the current CAML case study, the matched signature size was *m* = 18.

For neural network comparisons, we constructed a multilayer perceptron (MLP) classifier using TensorFlow. The architecture comprised an input layer corresponding to the feature subset size, followed by three fully connected hidden layers with 128, 64, and 32 units, respectively. All hidden layers used the rectified linear unit (ReLU) activation function, while the output layer used a sigmoid activation function for binary classification, defined as relapse versus non-relapse. The model was optimized using the Adam optimizer with a learning rate of 0.001 and binary cross-entropy loss. Training was conducted with a batch size of 32 for a maximum of 100 epochs, and early stopping with a patience of 3 epochs was applied within each training fold to reduce overfitting during training.

We also evaluated ensemble and gradient-based boosting strategies. Random forest (RF) was implemented with 100 decision trees, with feature importance assessed based on Gini impurity. eXtreme Gradient Boosting (XGBoost) was configured with a learning rate of 0.1, a maximum depth of 3, and 100 estimators. Standard gradient boosting (GB) and AdaBoost were implemented using default scikit-learn parameters to represent stage-wise ensemble techniques.

Baseline classifiers served as a fundamental reference for performance evaluation. These included an SVM with an RBF kernel, where the hyperparameters *C* and γ were optimized via grid search over a predefined parameter space (e.g., $C\in\{{10}^{-3},{10}^{-2},\ldots,{10}^{3}\}$, $\gamma\in\{{10}^{-3},{10}^{-2},\ldots,{10}^{3}\}$) using inner cross-validation within the training data. Logistic regression (LR) was applied with L2 regularization (Ridge) using default settings. k-nearest neighbors (KNN) was implemented with k = 5 neighbors using Euclidean distance. Gaussian naive Bayes (GNB) and decision tree (DT), using the CART algorithm with Gini impurity, were included to provide a comparative assessment across different inductive biases.

**Supplementary Note 7. Robustness analysis and topological bias control.**

**Expansion of the curated AML target reference set**

To evaluate the target-ranking performance of the TargetPrior framework and support bias-aware benchmarking, the pharmacological reference set was expanded to include nine AML-associated target references spanning clinically established, pharmacologically investigated, and biologically supported evidence tiers. The expanded benchmark set comprised AKT1, TOP2A, CCL2, RARA, FLT3, BCL2L2, MEN1, MCL1, and MDM2.

**Generation of data perturbations via repeated stratified splitting**

To evaluate the robustness of the EL-CAML framework against sample-level data perturbations beyond algorithmic convergence, we implemented a repeated stratified data-splitting protocol. The full CAML cohort was resampled across 30 independent iterations. In each iteration, the dataset was randomly divided into a training set and an internal test set using an approximate 80:20 ratio while preserving the relapse and non-relapse class distribution.

To preserve iteration-specific outputs and improve auditability, each iteration was stored in a separate output directory (folders 1–30) containing the corresponding perturbed training and test datasets. The entire EL-CAML pipeline, including feature subset selection, SVM hyperparameter tuning, and cross-validation, was independently re-executed from scratch within each perturbed training set.

**Co-expression-corrected stability analysis**

Let $S_{j}\subseteq\mathcal{F}$ denote the selected miRNA subset obtained from the *j*-th perturbation experiment, where $\mathcal{F}$ represents the complete candidate feature pool. In high-dimensional omics data, identical biological signals are often represented by highly correlated alternative biomarkers. To reduce potential underestimation of feature-set stability caused by such functional redundancy, a co-expression correction procedure was applied prior to stability evaluation.

Specifically, miRNAs exhibiting high transcriptomic correlation (Pearson’s |*r*| ≥ 0.6) were grouped into co-expression clusters. During evaluation, any selected miRNA was collapsed into the predefined representative feature of its respective cluster, defined operationally as the minimum feature index. The resulting co-expression-corrected feature subset was denoted as $\hat{S_{j}}$.

**Quantitative evaluation metrics**

Feature selection stability across the repeated perturbations was quantified by performing pairwise comparisons between all experiment pairs $\left( \hat{S_{i}},\hat{S_{j}} \right)$. Three complementary stability metrics were calculated: (1) Jaccard Index: $J\left( \hat{S_{i}},\hat{S_{j}} \right)=\frac{\left| \hat{S_{i}}\cap\hat{S_{j}} \right|}{\left| \hat{S_{i}}\cup\hat{S_{j}} \right|}$; (2) Overlap Coefficient: $O\left( \hat{S_{i}},\hat{S_{j}} \right)=\frac{\left| \hat{S_{i}}\cap\hat{S_{j}} \right|}{\min\left( \left| \hat{S_{i}} \right|,\left| \hat{S_{j}} \right| \right)}$; (3) Kuncheva Index: $KI\left( \hat{S_{i}},\hat{S_{j}} \right)=\frac{rn-k^{2}}{k\left( n-k \right)}$, where $r=\left| \hat{S_{i}}\cap\hat{S_{j}} \right|$, *k* denotes the selected feature-set size, and *n* is the total candidate feature pool size. When the corrected subsets differed in size, *k* was defined as the mean selected feature-set size for the compared pair. The Kuncheva Index specifically corrects for the probability of subsets overlapping purely by chance.

To systematically benchmark target-reference retrieval performance within the inferred network, candidate genes were ranked according to the selected centrality-based ranking metric. Prioritization performance was evaluated across multiple top-K thresholds (K = 10, 20, 50, 100) using the following metrics: (1) Precision@K: the proportion of top-K prioritized candidate genes belonging to the AML target reference set; (2) Recall@K: the proportion of all AML target reference genes retrieved within the top-K ranks; (3) mean reciprocal rank (MRR): the mean reciprocal rank ($1/\text{ran}\text{k}_{\text{i}}$) across AML target reference genes, assigning greater weight to reference genes ranked near the top of the list; (4) Mean Rank: the arithmetic mean of the absolute network ranks of the retrieved AML target reference genes; and (5) Target coverage count: the number of gold-standard targets successfully identified within the constructed regulatory network.

**Statistical validation and degree-quantile-matched permutation for topological bias control**

To evaluate whether the prioritization results reflected the TargetPrior-derived miRNA anchors rather than network degree, annotation density, or literature-driven hub bias in miRTarBase, three complementary statistical frameworks were implemented: (1) Hypergeometric test: a hypergeometric test evaluating the probability of observing the overlap between the AML target reference genes and the top-*K* ranked candidates under random selection, given the total network size; (2) Mann–Whitney U test: a non-parametric Mann–Whitney U test evaluating whether the AML target reference genes received higher centrality-based ranking scores, or equivalently earlier ranks, than background network genes; and (3) degree-quantile-matched permutation analysis: an empirical permutation protocol used to control for degree-associated literature bias. All network nodes were stratified into topological bins based on the log_2_-transformed degree distribution. A Monte Carlo procedure with 1,000 independent permutations was performed to generate degree-matched random reference sets by sampling nodes from the same empirical degree quantile bins as the observed AML target reference genes. The empirical *P*-value represents the probability that degree-matched random reference sets achieved a retrieval or centrality score at least as strong as the observed AML target reference set. A *P*-value < 0.05 was interpreted as supporting target-reference enrichment beyond degree-matched random expectation, rather than as definitive evidence of biological causality or freedom from all literature bias.

**Supplementary Note 8. Scalability and proposed cross-disease application benchmarks of the TargetPrior framework.**

TargetPrior is designed as a modular computational protocol that can be adapted to disease-specific transcriptomic datasets. Its applicability requires standardized transcriptomic input matrices (e.g., TARGET data matrices) and integration with curated drug–target (e.g., DrugBank) knowledge bases. In this section, we outline potential cross-disease application scenarios to illustrate how the framework may be extended beyond CAML.

For example, in high-risk neuroblastoma (TARGET-NBL), where the standard-of-care involves Isotretinoin (validated in trial COG-ANBL0532, NCT00567567), the TargetPrior framework could in principle be applied to evaluate regulatory modules involving RARA, RXRB, and RARG within the inferred network structure (Isotretinoin in DrugBank: DB00982, Etretinate in DrugBank: DB00926). Similarly, for osteosarcoma (TARGET-OS), where the MAP regimen includes methotrexate (validated in trial EURAMOS-1, NCT00134030), the framework could be adapted to assess centrality patterns of TYMS, ATIC, and DHFR (Methotrexate in DrugBank: DB00563) within the corresponding disease-specific transcriptomic network. These scenarios illustrate the potential transferability of TargetPrior beyond leukemia-specific applications. However, they do not constitute empirical cross-disease validation and should be interpreted as proposed application scenarios for future benchmarking across cancer transcriptomic datasets.

**Supplementary Figure 1. Sensitivity analysis supporting the selection of the transcriptomic filtering threshold (*τ_fc_* = 1.3).** (A) The sensitivity analysis plot illustrates the reduction in the number of identified differentially expressed genes (DEGs) as the |log_2_ FC| threshold increases. The 1.3 threshold (red line) serves as an elbow point, filtering out ~50% of low-effect transcriptomic variations compared to the conventional threshold of 1.0. (B) The effect size distribution bar plot compares the average effect size (mean |log_2_ FC|) of genes selected at different thresholds. The 1.3 threshold yields a significantly higher mean effect size (1.79) compared to 1.0 (1.48), indicating selection of genes with larger expression differences. (C) The volcano plot highlights the distribution of DEGs. The strict 1.3 cutoff (red vertical lines) effectively segregates the high-confidence dysregulated genes from the dense cluster of moderate variations (gray points between blue and red lines), thereby reducing network density for downstream topological analysis.

**Supplementary Figure 2. Dual-axis plot illustrating the stability–performance trade-off across candidate feature subset sizes**. The bar chart (left *y*-axis, blue) represents the algorithmic stability, defined as the subset-level stability score computed across 30 independent IBCGA runs. The line chart (right *y*-axis, green) shows the mean 5-fold cross-validation (5-CV) Matthews correlation coefficient. The 18-miRNA parsimony signature (*S_cons_*) represents the selected stability–performance balance point, where predictive fidelity maximizes before a marked decrease in subset stability.

**Supplementary Figure 3. Scatter plot illustrating the stability–performance trade-off for the selection of the 18-miRNA**  **parsimony signature (*S_cons_*).** Each data point represents a candidate model evaluated at a specific feature count. The *S_cons_* (red star) is located in the upper-right region of the stability–performance space, indicating a favorable balance between subset-level stability and predictive performance.

**Supplementary Figure 4. Reliability diagrams and probability calibration curves of the EL-CAML continuous risk-scoring framework on the independent test cohort.** The calibration plot visualizes the relationship between the predicted relapse-risk scores partitioned into 10 empirical bins on the x-axis and the observed fractional relapse outcomes within the cohort on the y-axis. The diagonal dashed line represents the reference line for perfect probability calibration. The orange line denotes the raw, uncalibrated piecewise-linear normalized risk score. The green and red lines illustrate post hoc parametric Platt scaling and non-parametric isotonic regression mappings, respectively.

**Supplementary Figure 5. Box plots illustrating the differential expression levels of the parsimony signature (*S_cons_*) between CAML patients and healthy controls.** Statistical significance between the two cohorts was evaluated using the Wilcoxon rank-sum test (* P ≤ 0.05, ** P ≤ 0.01, and *** P ≤ 0.001).

**Supplementary Figure 6. Receiver operating characteristic (ROC) curves evaluating the diagnostic discrimination of the 18-miRNA parsimony signature (*S_cons_*) in an independent cohort.** The ROC analysis evaluates the ability of the signature to distinguish CAML patients from healthy controls. The corresponding area under the curve (AUC) values are annotated within the plots.

**Supplementary Figure 7. Kaplan–Meier (KM) overall survival analysis of individual miRNAs from the 18-miRNA parsimony signature (*S_cons_*).** KM curves show the survival associations of the 3rd, 4th, 5th, 6th, 12th, and 16th highest-ranked miRNAs, ranked according to their main effect difference (MED) scores in Table 1. Statistical significance of overall survival differences between patient strata was evaluated using the log-rank test.

**Supplementary Figure 8. Receiver operating characteristic (ROC) curves evaluating the univariate predictive capacity of individual miRNAs.**

**Supplementary Figure 9. Evaluation of feature stability across 30 independent EL-CAML runs.** The bar chart displays the selection count on the *y*-axis for candidate miRNAs ranked by their selection stability. (A) The red bars highlight the 18-miRNA parsimony signature (*S_cons_*). A marked decrease in selection count is observed after the 25th feature. Statistical comparison using the Wilcoxon rank-sum test indicates that the TargetPrior-selected signature is significantly distinct from low-frequency background features (P < 0.001), supporting the selection-frequency threshold (≥ 9 runs, corresponding to *f_i_* ≥ 0.30). The mean selection counts (± standard deviation [SD]) are as follows: the 18-miRNA *S_cons_*: 10.55 ± 6.47, ten complementary biomarkers (*S_comp_*): 13.20 ± 4.44, and low-frequency background features (feature number = 139): 2.39 ± 2.36.

**Supplementary Figure 10.** **Differential expression profiles of the EL-derived miRNA signatures in an independent cell-sorted pediatric AML dataset (GSE196886).** The bar chart illustrates the log_2_ fold change (log_2_FC) of the identified miRNAs in leukemic stem cells (LSCs) relative to normal hematopoietic stem cells (HSCs). The horizontal solid black line represents the baseline of equal expression (log_2_FC = 0), whereas the red dashed lines delineate a reference magnitude threshold of |log_2_FC| = 1. The evaluated miRNAs on the x-axis are categorically grouped to reflect the two structural components of the TargetPrior miRNA anchor set: the 18-miRNA parsimony signature (*S_cons_*, denoted by the yellow bars) and the complementary biomarkers (*S_comp_*, denoted by the green bars).
